# MqsR is a noncanonical microbial RNase toxin that is inhibited by antitoxin MqsA *via* steric blockage of substrate binding

**DOI:** 10.1016/j.jbc.2022.102535

**Published:** 2022-09-24

**Authors:** Victor Yu, Erik Ronzone, Dana Lord, Wolfgang Peti, Rebecca Page

**Affiliations:** 1 Department of Cell Biology, University of Connecticut Health Center, Farmington, Connecticut, USA; 2 Department of Molecular Biology, Cell Biology and Biochemistry, Brown University, Providence, Rhode Island, USA; 3 Department of Molecular Biology and Biophysics, University of Connecticut Health Center, Farmington, Connecticut, USA

**Keywords:** Toxin-antitoxin, RNase, MqsR, MqsA, NMR spectroscopy, CSP, chemical shift perturbation, Gdn-HCl, guanidine hydrochloride, HSQC, heteronuclear single quantum correlation, TA, toxin:antitoxin

## Abstract

The MqsRA toxin-antitoxin system is a component of the *Escherichia coli* stress response. Free MqsR, a ribonuclease, cleaves mRNAs containing a 5′-GC-3′ sequence causing a global shutdown of translation and the cell to enter a state of dormancy. Despite a general understanding of MqsR function, the molecular mechanism(s) by which MqsR binds and cleaves RNA and how one or more of these activities is inhibited by its cognate antitoxin MqsA is still poorly understood. Here, we used NMR spectroscopy coupled with mRNA cleavage assays to identify the molecular mechanism of MqsR substrate recognition and the MqsR residues that are essential for its catalytic activity. We show that MqsR preferentially binds substrates that contain purines in the −2 and −1 position relative to the MqsR consensus cleavage sequence and that two residues of MqsR, Tyr81, and Lys56 are strictly required for mRNA cleavage. We also show that MqsA inhibits MqsR activity by sterically blocking mRNA substrates from binding while leaving the active site fully accessible to mononucleotides. Together, these data identify the residues of MqsR that mediate RNA cleavage and reveal a novel mechanism that regulates MqsR substrate specificity.

Bacteria have evolved multiple, diverse mechanisms to effectively respond to rapidly changing environmental conditions (1, 2, 3, 4). This includes the existence of distinct gene pairs known as toxin:antitoxin (TA) systems, small mobile genetic elements composed of a toxin, which causes growth arrest by interfering with an essential cellular process, and a cognate antitoxin, which neutralizes the toxin activity during normal growth conditions (5). Although the environmental conditions that activate distinct TA systems are still poorly understood, the result is an excess of toxin, whose activity leads to rapid growth arrest and dormancy (6).

Type II TA systems are the largest and best-studied class of TA system, with many type II TA loci identified in most free-living bacteria, such as *Escherichia coli* (7, 8). In type II TA systems, both the antitoxin and toxin genes code for proteins (5). Typically, type II antitoxins have two domains, a DNA-binding domain (to bind the promoters of their own operon to repress transcription (9)) and a second domain that binds and inhibits the activity of their cognate protein toxin. Toxins typically either inhibit replication (*i.e.*, by inhibiting DNA gyrase) (10) or translation (*i.e.*, by cleaving mRNA) (11, 12). Furthermore, some inactivate ribosome elongation factors (13). Many type II toxins are endoribonucleases (RNases) (14) and have been shown to function as either ribosome-dependent (RelE (15), YoeB (16), YhaV (17), HigB (18)) or ribosome-independent (MazF (11) and MqsR (19, 20); the latter of which is more similar to canonical microbial RNases such as RNase SA (21)). These classifications are consistent with recent RNAseq studies that defined elements of their cleavage specificities, with ribosome-dependent RNases exhibiting a strong bias for cleaving mRNAs at the 5′ end of coding regions and ribosome-independent RNases lacking this bias (22). Antitoxin-mediated inhibition of their cognate toxins is typically achieved by binding and blocking or distorting the active site and/or blocking ribosome binding (*i.e.*, for ribosome-dependent RNases) (21, 23, 24, 25). Despite these advances, we still lack an understanding of the additional substrate elements that contribute to toxin binding and antitoxin-mediated inhibition of toxin activity.

One toxin whose mechanisms of mRNA cleavage and inhibition by its cognate antitoxin are not fully defined is that of MqsR, an *E. coli* RNase (MqsA is its cognate antitoxin). MqsR adopts a RelE-like fold (21) and, like RelE, has a moderate preference for cleaving transcripts near the 5′ end (22). However, unlike RelE, it does not require the ribosome for activity (19, 22). Conversely, unlike most known ribosome-independent RNases and their microbial RNase homologs, MqsR also does not have a conserved histidine and glutamic acid that function as a catalytic acid and base, respectively (26). Furthermore, while it is established that MqsR targets a broad range of mRNA transcripts containing the consensus sequence 5′-GC(U/A)-3′ or 5′-GC-3′ (20, 22, 27), additional substrate elements that enhance MqsR-mediated degradation are still undefined. Finally, the structure of the MqsR–MqsA complex defined the location of the MqsA interaction site on MqsR, revealing that the putative MqsR active site is solvent accessible in the complex (21). Thus, a molecular understanding of how MqsA prevents MqsR-mediated cleavage of mRNA substrates has remained elusive.

Here, we used biomolecular NMR spectroscopy, coupled with mRNA cleavage assays, to answer these outstanding questions. NMR-based chemical shift perturbation (CSP) measurements using both mononucleotides and mRNA/DNA hybrid substrate analogs showed that MqsR preferentially binds longer substrates that include purines at the −2 and −1 position relative to the consensus cleavage sequence. Further, the data also showed that multiple MqsR residues contribute to substrate binding. To distinguish the role of these MqsR residues in substrate recruitment and for the catalytic mechanism of MqsR, we performed mutagenesis coupled with RNase cleavage assays. These data identified multiple residues important for MqsR-mediated mRNA cleavage, with two, Lys56 and Tyr81, being essential for catalysis. Finally, using CSP data, we showed that MqsA inhibits MqsR activity not by binding and/or distorting the active site but instead by blocking substrate binding to the active site. Together, this work provides a molecular understanding of how MqsR, a noncanonical microbial RNase toxin, cleaves its mRNA substrates and how this activity is potently neutralized by its cognate antitoxin MqsA.

## Results

### Sequence-specific backbone assignment of MqsR

**Figure 1:**
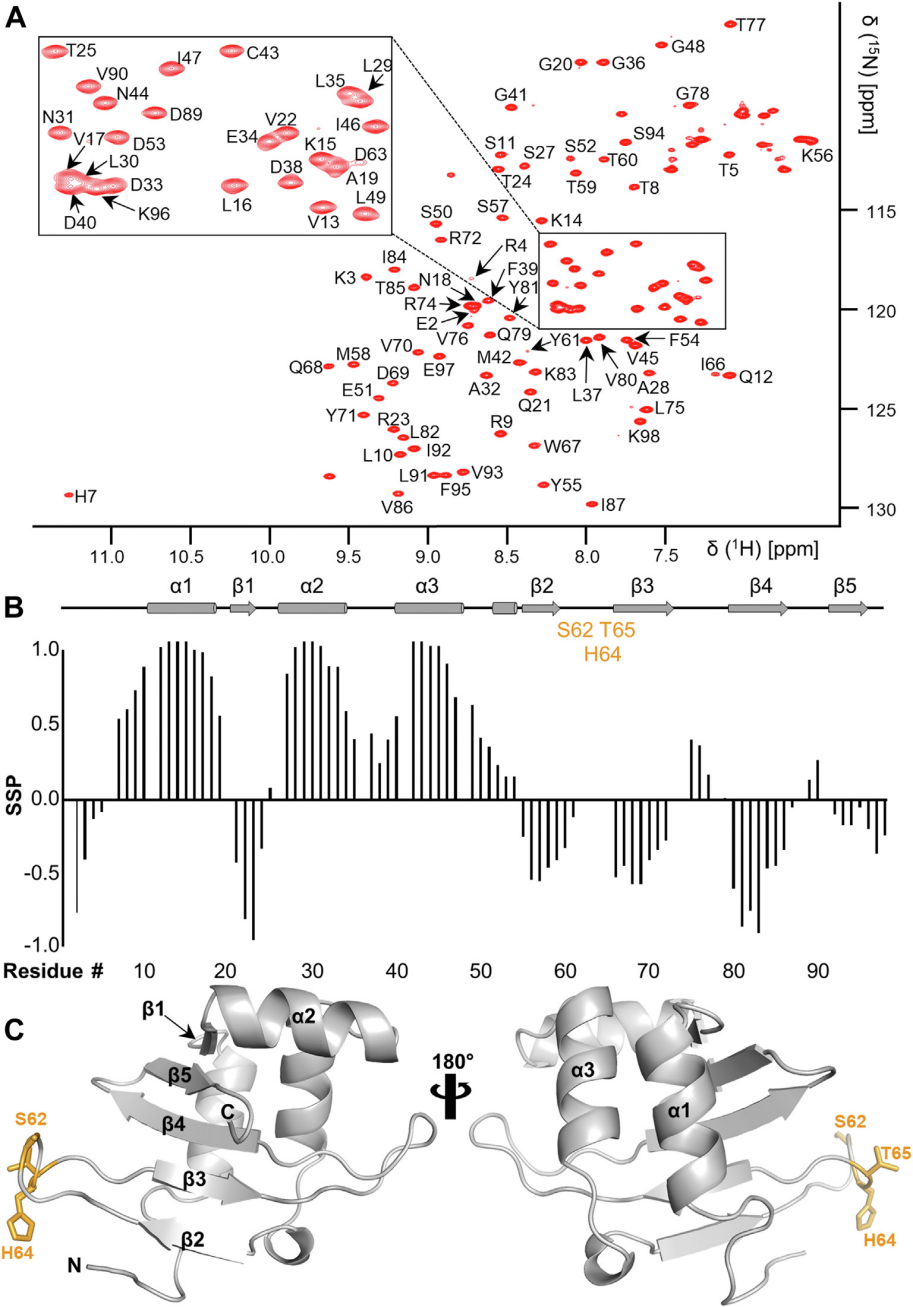
**MqsR sequence specific backbone assignment.***A*, annotated 2D [^1^H,^15^N] HSQC spectrum of MqsR. *B*, secondary structure propensities (SSP) of MqsR. α-helices and β-strands present in the MqsR crystal structure (PDB ID: 3HI2) are shown as *cylinders* and *arrows*, respectively. *C*, crystal structure of MqsR from the same complex shown as a *cartoon*. Residues in the β2-β3 loop whose peaks are either not assigned or not present in the NMR spectrum are shown as *sticks* and colored *light orange* (missing). HSQC, heteronuclear single quantum coherence.

The 2D [^1^H,^15^N] heteronuclear single quantum coherence (HSQC) spectrum of MqsR (98 residues, 11.2 kDa, Fig. 1*A*) is consistent with a well-folded protein, as expected from the MqsR structure that was determined by X-ray crystallography (21). To probe the interaction of MqsR with substrates and proteins in solution, we completed the MqsR sequence-specific backbone assignment. Secondary structure propensity of Cα and Cβ chemical shifts (28) agrees well with the secondary structure of the MqsR crystal structure (Fig. 1*B*). Ninety NH cross peaks (of 96 possible) were assigned (94%). MqsR residues Ser62, His64, and Thr65, which belong to the β2 and β3 connecting loop, were not assigned (Fig. 1*C*). Furthermore, neighboring residues Tyr61, Glu63, and Ile66 have weak cross-peak intensities in the 2D [^1^H,^15^N] HSQC spectrum of MqsR, suggesting that the missing loop residues are broadened beyond detection as a result of sampling multiple conformations at a μs/ms intermediate timescale.

### The MqsR nucleotide substrate-binding site is centered on Tyr81

**Figure 2:**
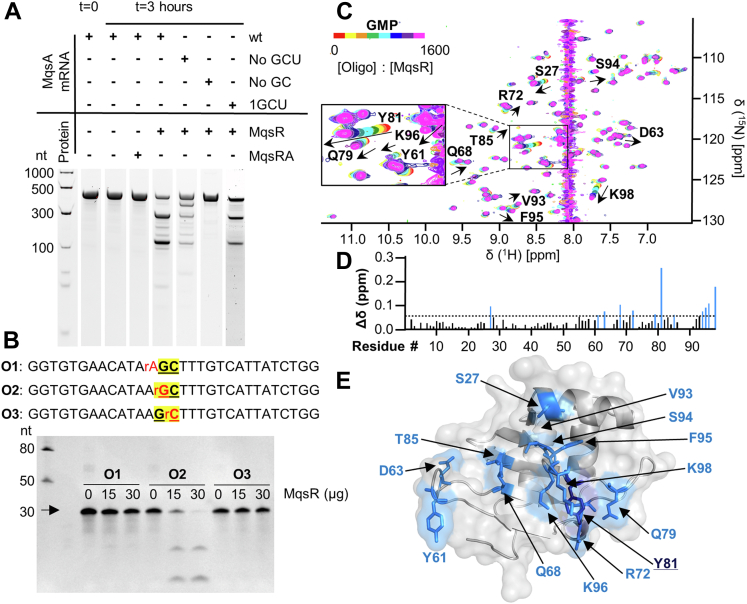
**MqsR is a guanosine-directed RNase that requires GC sequences for cleavage.***A*, mRNA cleavage assays of wt MqsA mRNA and variants (no GCU, lacks all GCU sequences; no GC, lacks all GC sequences; mRNA substrate sequences are listed in Table S1) with or without MqsR protein or MqsR:MqsA protein complex. *B*, DNA/RNA hybrid oligos (r indicates oxynucleotide; remaining nucleic acids are deoxy) used for cleavage assays in the presence of 0, 15, 30 μg MqsR. The single cleavable nucleotide is indicated in *red* and the GC cleavage site is in *bold* and *underlined*. *C*, overlay of the 2D [^1^H,^15^N] HSQC spectrum of ^15^N-labeled MqsR titrated with increasing concentrations of GMP. Peaks experiencing CSPs due to increasing GMP concentrations are indicated by *arrows* and labeled. *D*, CSPs for MqsR:GMP plotted by residue number. Residues with significant CSPs (>1σ_0_; indicated by a *dotted line*) are shown in *blue*. *E*, MqsR crystal structure with residues that exhibit significant CSPs (>1σ_0_) shown as *sticks*, colored *blue*, and labeled; Y81, which exhibits the largest CSP, is shown in *dark blue*. These residues were used to calculate a global binding K_D_ in Table 1. CSP, chemical shift perturbation; HSQC, heteronuclear single quantum correlation.

MqsR cleaves single stranded 5′-GC(U/A)-3′ mRNA sequences (27, 29). Furthermore, recent transcriptome sequence experiments refined the MqsR specificity cleavage sequence to 5′-GC-3′ (22, 30), with MqsR specifically targeting the guanosine moiety of GC motifs for cleavage (14, 20, 27). We confirmed this using *in vitro* cleavage assays with purified MqsR and distinct mRNA substrates, including wt-MqsA mRNA and variants which lack all 5′-GCU-3′ or 5′-GC-3′ sequences (Fig. 2*A* and Table S1). *In vitro* cleavage assays using three RNA/DNA oligos containing a single cleavable ribonucleotide showed that MqsR targets G nucleosides for cleavage (Fig. 2*B*).

To identify MqsR residues that mediate 5′-GC-3′ sequence binding and cleavage, we titrated GMP into ^15^N-labeled MqsR and followed the resulting CSPs using 2D [^1^H,^15^N] HSQC spectra. CSPs report on a change of the local environment of the H^N^ reporter, either *via* a direct binding interaction with the titrant and/or an indirect conformational change (high ratios of MqsR:GMP [1:1600] were required for these titrations due to its mM binding affinity; Fig. 2*C,* Table 1) (31). Significant CSPs were determined by calculating the SD of all CSPs (σ), excluding any CSPs greater than 3σ above the average, recalculating σ as a corrected σ_0_, and then iteratively removing CSPs above 3σ_0_ until no more CSPs could be removed. CSPs above ≥1σ_0_ were then selected as the threshold (32, 33). These CSPs were observed for 13 MqsR residues: Ser27, Tyr61, Asp63, Qln68, Arg72, Gln79, Tyr81, Thr85, Val93, Ser94, Phe95, Lys96, and Lys98 (Fig. 2*D*; ^1^H aromatic NMR signals of GMP at high concentrations interfered with MqsR signals). Mapping these residues onto MqsR (Fig. 2*E*) identified the interface used for mononucleotide binding. Most MqsR residues that exhibited CSPs were clustered on the central MqsR β-sheet, a location consistent with the active sites of other homologous RNases (Fig. S1). Two additional residues in the β2/β3 loop, Tyr61 and Asp63, also exhibit CSPs, suggesting that this loop region may also be involved in binding mRNA substrates.

To determine if specific MqsR residues are necessary for guanosine binding, we also titrated UMP into MqsR (Fig. S2). MqsR also showed CSPs with UMP; however even higher MqsR:UMP ratios were required, indicating that the interaction is weaker. Five MqsR residues (Ser27, Tyr61, Tyr81, Phe95, and Lys98) exhibited CSPs with both UMP and GMP, suggesting these residues define the core binding pocket of MqsR. Using the CSPs ≥ 1σ_0_ (for GMP and UMP, respectively) to calculate residue specific and global average binding affinities (K_D_; Table S2), we determined that GMP binds MqsR ∼2-fold stronger than UMP (GMP K_D_: 30 ± 18 mM; UMP K_D_: 54 ± 30 mM; Table 1) (32).

**Table 1:** Global-binding affinities of MqsR with noncleavable substrate analogs

Substrate	Nickname	Global K_D_ (mM)
Mononucleotides		
GMP		30 ± 18
UMP		54 ± 30
Vary substrate length		
AdGCUA	Oligo-A	1.5 ± 1.0
AAdGCUA	Oligo-AA	0.22 ± 0.06
AAAdGCUA	Oligo-AAA	0.15 ± 0.05
Vary the −2 nucleotide		
AAdGCUA	Oligo-AA	0.22 ± 0.06
GAdGCUA	Oligo-GA	0.23 ± 0.05
UAdGCUA	Oligo-UA	1.12 ± 0.50
CAdGCUA	Oligo-CA	2.17 ± 0.80
Vary the −1 nucleotide		
AAdGCUA	Oligo-AA	0.22 ± 0.06
AGdGCUA	Oligo-AG	0.34 ± 0.10
AUdGCUA	Oligo-AU	0.58 ± 0.20
ACdGCUA	Oligo-AC	0.65 ± 0.30

### MqsR preferentially binds longer mRNA substrates

**Figure 3:**
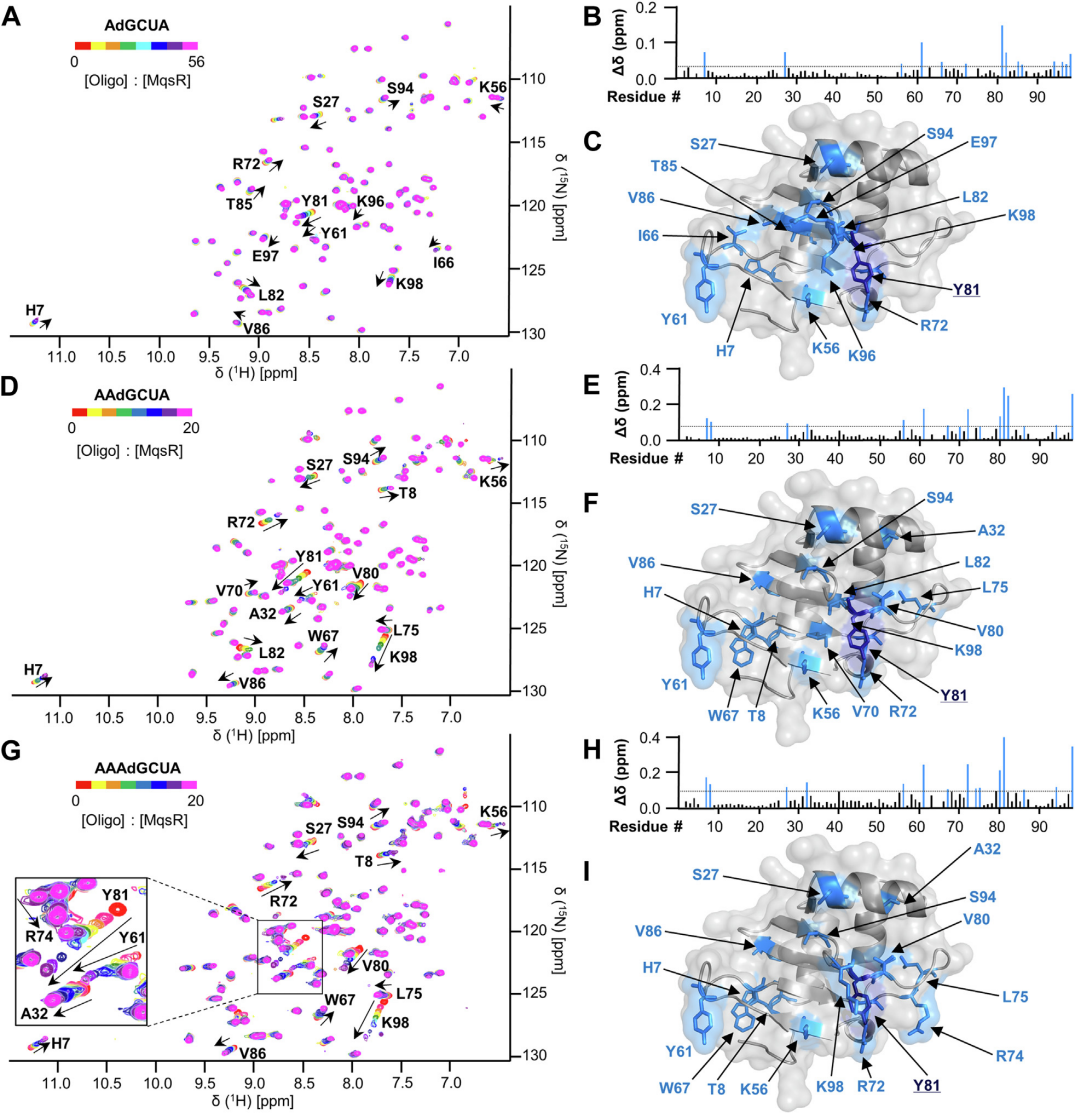
**MqsR interacts more strongly with longer mRNA substrates.***A*, overlay of the 2D [^1^H,^15^N] HSQC spectra of ^15^N-labeled MqsR with increasing concentrations of the nonhydrolyzable mRNA substrate 5′-AdGCUA-3′ (d indicates the base is deoxy; Oligo-A). Peaks exhibiting CSPs due to the presence of the mRNA substrate are indicated by *arrows* and labeled. *B*, CSPs for MqsR:oligo-A. Residues with significant CSPs (>1σ_0_; indicated by a *dotted line*) are indicated in *blue*. *C*, MqsR crystal structure with residues that exhibit significant CSPs (>1σ_0_) shown as *sticks*, colored *blue*, and labeled; Y81, which exhibits the largest CSP, is shown in *dark blue*. *D–F*, same as (*A*), (*B*), and (*C*), respectively but with nonhydrolyzable mRNA substrate 5′-AAdGCUA-3′ (Oligo-AA). *G–I*, same as (*A*), (*B*), and (*C*), respectively but with nonhydrolyzable mRNA substrate 5′-AAAdGCUA-3′ (Oligo-AAA). CSP, chemical shift perturbation; HSQC, heteronuclear single quantum coherence.

To explore whether MqsR interacts with residues outside the canonical 5′-GC-3′ cleavage site, we also titrated MqsR with a series of noncleavable mRNA substrates (containing a deoxyguanosine, dG, to prevent MqsR cleavage). All sequences contained the invariant 5′-dGCUA-3′ sequence yet differed in length (5- to 7-mers) and nucleotide identity outside the invariant sequence (Table 1), allowing the effects of substrate length and sequence on MqsR binding to be determined. We first titrated increasing ratios of 5′-AdGCUA-3′ into ^15^N-labeled MqsR and followed the resulting CSPs using 2D [^1^H,^15^N] HSQC spectra. Fourteen peaks showed significant CSPs (≥1σ_0_), including residues His7, Ser27, Lys56, Tyr61, Ile66, Arg72, Tyr81, Leu82, Thr85, Val86, Ser94, Lys96, Glu97, and Lys98 (Fig. 3, *A*–*C*; Table S3). Four of the five residues that we identified as nondiscriminate interactors (Ser27, Tyr61, Tyr81, and Lys98) in the GMP/UMP experiments exhibited large CSPs. Analyzing all peaks with CSPs ≥1σ_0_ results in a global average K_D_ of 1.5 ± 1.0 mM, ∼20-fold tighter than GMP (Table 1).

Next, we tested if extending the noncleavable substrate in the 5′ direction, 5′-AAdGCUA-3′, influences MqsR binding (Fig. 3, *D–F*). As expected, a common core set of residues (His7, Ser27, Lys56, Tyr61, Arg72, Tyr81, Leu82, Val86, Ser94, and Lys98) interact with both 5′-AdGCUA-3′ (Oligo-A) and 5′-AAdGCUA-3′ (Oligo-AA) (Table S3) demonstrating that these substrates bind the same interface of MqsR. However, the CSPs of these residues are far larger for the 6-mer than the 5-mer, demonstrating that the addition of a nucleotide at the −2 position enhances the substrate interaction with those residues of MqsR. Consistent with this, the global K_D_ also decreased by ∼7-fold to 0.22 ± 0.06 mM (Table 1). In contrast, extending the substrate by another nucleotide (5′-AAAdGCUA-3′, Oligo-AAA; Fig. 2, *G–I*) resulted in a nearly identical K_D_ (0.15 ± 0.05 mM), demonstrating that an additional nucleotide at the -3 position does not significantly enhance binding. These data show that substrate engagement is enhanced by nucleotides outside the GCU recognition sequence and, particularly, that a nucleotide located upstream (5′) at the −2 position of this sequence facilitates MqsR binding.

### Purines, but not pyrimidines, enhance MqsR substrate binding

We next used NMR-based CSP studies to determine if the observed enhancement of binding is sequence specific or, alternatively, if the addition of any nucleotide upstream of the consensus sequence enhances affinity. Thus, we tested the interaction of substrates that differed in the nucleotide identity at the −2 position relative to the consensus GCU sequence (*i.e.*, Oligo-AA; 5′-GAdGCUA-3′, Oligo-GA; 5′-UAdGCUA-3′, Oligo-UA; or 5′-CAdGCUA-3′, Oligo-CA). The data show that purines in the −2 position (Oligo-GA and Oligo-AA) bind MqsR ∼5- to 10-fold more tightly than pyrimidines (Oligo-UA and Oligo-CA; Table 1 and Fig. S3), despite binding essentially the same MqsR residues. We also tested if the identity of the nucleotide immediately 5′ of the GCU sequence (the −1 position) also affected substrate binding (substrates, Oligo-AA; 5′-AGdGCUA-3′, Oligo-AG; 5′-ACdGCUA-3′, Oligo-AC; 5′-AUdGCUA-3′, Oligo-AU). NMR-based CSP analysis showed that the identity of the nucleotide at the −1 position is less important for the interaction with MqsR (Table 1 and Fig. S4) but that purines (Oligo-AA and Oligo-AG) are still slightly preferred over pyrimidines (Oligo-AC and Oligo-AU). However, the differences in the K_D_ values between the substrates was much smaller than for the −2 position (∼3-fold *versus* ∼10-fold). Together, these data demonstrate that MqsR prefers the nucleotides in the order A > G >>> U > C in both the −2 and −1 nucleotide position relative to the consensus GCU sequence.

### mRNA substrates bind MqsR along a cleft extending from the β2-β3 loop to the active site

**Figure 4:**
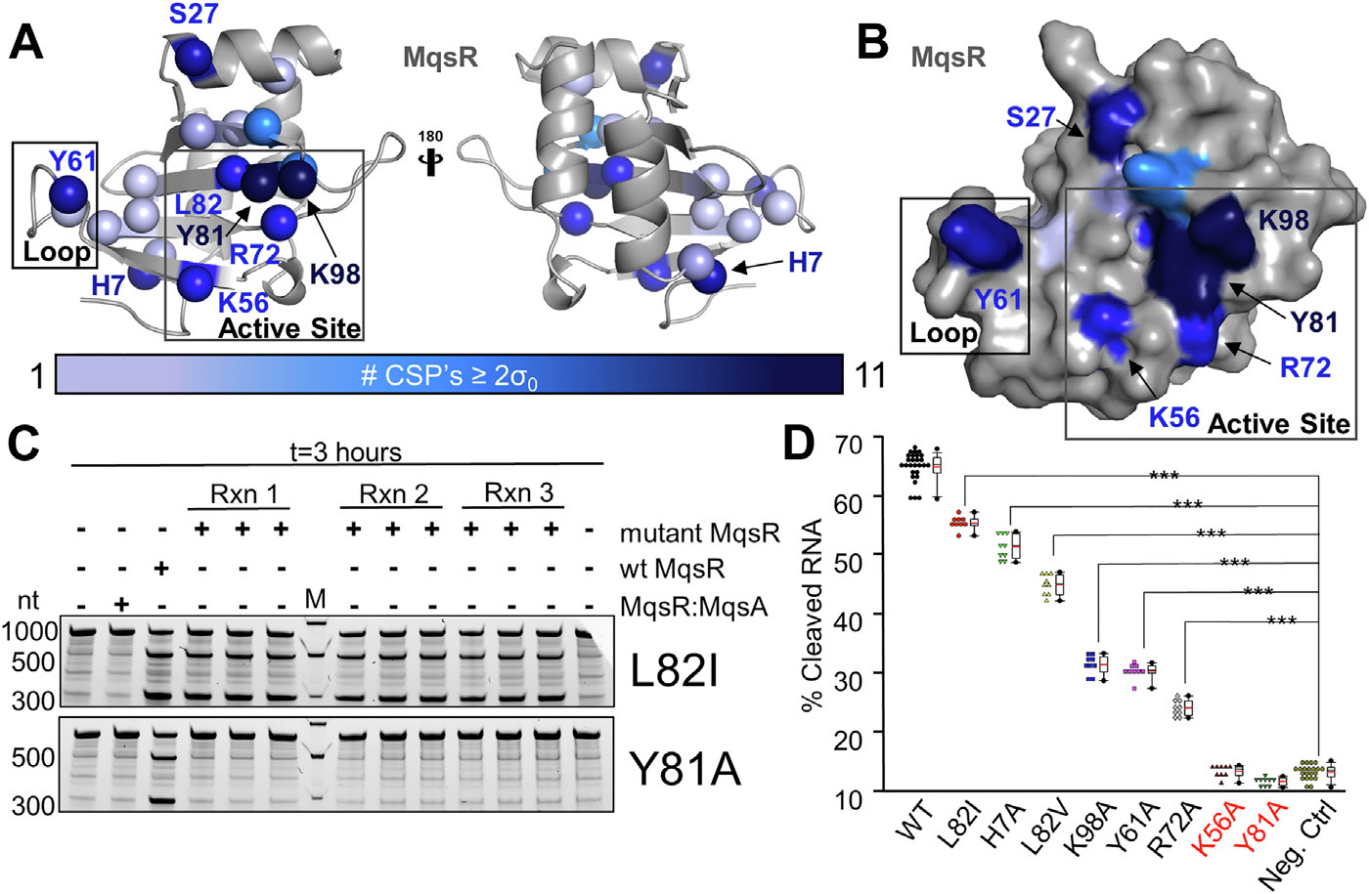
**mRNA substrates bind MqsR *via* an extensive surface.***A*, MqsR residues that exhibit CSPs >2σ_0_ in titrations with mRNA substrates are mapped onto MqsR and shown as *balls*. Residues colored by the number of titrations in which the residue experienced a CSP >2σ_0_ (11 distinct titrations performed with mononucleotides or nonhydrolyzable mRNA substrates). *B*, same as (*A*), except MqsR shown as a surface. *C*, representative cleavage assay data of the most (L82I) and the least active (Y81A) MqsR mutants using a substrate with a singular preferred 5′-AAGCU-3′ site (1GCU MqsA RNA). Substrate sequence in Table S1. All cleavage assays shown in Fig. S5. All mutants are assayed in three replicate reactions and each reaction is ran in triplicate yielding an n = 9 of each mutant. M, nt standards. *D*, quantified cleavage activity of mutants. A standard box plot is provided next to each mutant. The *red* mark represents the average value. ANOVA analysis indicates that Lys56 and Tyr81(*red*) are inactive. CSP, chemical shift perturbation.

In addition to classifying the extent to which different substrates bind MqsR, our NMR CSP titration data also identified which MqsR residues interact most strongly and most often with substrates, allowing us to determine which residues contribute to substrate affinity. Eighteen residues exhibited a CSP ≥ 2σ_0_ in at least one of the 11 NMR titrations performed (Fig. 4, *A* and *B* and Table S4). Ten of these residues experienced a significant CSP in six or fewer titrations, indicating that these residues are likely not the primary contributors to substrate binding (for example, while Ala32 exhibits significant CSPs in two titrations, it is completely solvent inaccessible indicating that the Ala32 CSPs are the result of indirect effects instead of a direct substrate interaction). In contrast, the remaining eight residues had significant CSPs in eight or more titrations and thus are likely important for substrate binding. These substrate-binding residues can be broadly grouped into four distinct elements. The largest cluster of MqsR residues is centered on Tyr81 and Lys98 (two residues that exhibited the largest CSPs in nearly all titrations) and includes MqsR residues Lys56, Arg72, and Leu82. This cluster lies along the β-sheet of MqsR and overlaps closely with the positions of catalytic His/Glu motif residues in homologous RNases (Fig. S1), suggesting this cluster of residues defines the active site of MqsR. Outside of this active site region, the remaining three residues are found on separate elements, including Tyr61 which is part of the β2-β3 loop, Ser27 on the α2 helix, and His7 in the MqsR N-terminus.

### Lys56 and Tyr81 play critical roles in the catalytic mechanism of MqsR

Our CSP experiments identified eight MqsR residues that are critical for mRNA binding (Table S3; residues that experienced CSPs with all substrates). To determine if they also are critical for MqsR-mediated mRNA cleavage, we used mutagenesis coupled with mRNA cleavage assays. First, for each of the eight MqsR residues, we created an Ala variant (for Leu82, whose sidechain is buried in the hydrophobic core (34), we generated L82I and L82V variants) and confirmed their folding status using differential scanning fluorimetry assays (Table S5). Only MqsR_S27A_ did not refold properly and was thus excluded from further study. The remaining eight MqsR variants were stable and well folded. *In vitro* mRNA cleavage assays were then performed with the purified MqsR mutants using an MqsA substrate with a single 5′-AAGCUA-3′ cleavage site (assays were performed at both 37 °C, Figs. 4*C* and S5 and 25 °C, Fig. S6; cleavage results at both temperatures were essentially relatively identical).

As expected from our NMR data, all MqsR variants exhibited reduced activity compared to wt MqsR (Fig. 4*D*). However, the extent of activity loss differed for each variant, with three variants exhibiting moderate cleavage (H7A, L82V, L82I), three variants exhibiting weak cleavage (K98A, Y61A, and R72A), and two resulting in no cleavage (K56A and Y81A). Importantly, MqsR variants K56A and Y81A are catalytically inactive (statistically indistinguishable from the mRNA alone negative controls), demonstrating they are key mediators of catalysis in MqsR. Consistent with this result, overlaying these residues of MqsR with other well-studied RNases shows that both Lys56 and Tyr81 are located in positions similar to the conserved Glu/His catalytic pair of other RNases (Fig. S1) (16, 34, 35, 36). The remaining residues (Arg72, Lys98, Tyr61, and His7), which interact with mRNA but whose mutation to Ala has a smaller impact on activity, likely have only a limited role in catalysis.

### The mechanism of toxin inhibition by its cognate antitoxin MqsA

Distinct antitoxins use different mechanisms to inhibit their cognate toxins, including blocking ribosome binding (a step essential for ribosome-dependent RNases for mRNA cleavage, *i.e.*, RelE/B; (24) or by blocking the toxin active site (*e.g.* VapX/D (37); MazE/F (25))), among others. How MqsA inhibits MqsR activity is still poorly understood. These data, coupled with the previously determined MqsR:MqsA structure (21), show that MqsA binds MqsR *via* an interface that is distal from the now experimentally determined catalytic site (Fig. S7*A*). This demonstrates that MqsA does not bind and block the toxin active site. To identify how MqsA inhibits MqsR, we again used NMR spectroscopy. First, we titrated the unlabeled N-terminal toxin-binding domain of MqsA (residues 1–76; hereafter referred to as MqsA_1-76_) into ^15^N-labeled MqsR. As expected for a very tight interaction (K_D_ < 1 nM) (19), sub-stoichiometric amounts of MqsA_1-76_ led to slow exchange, nearly doubling the number of observed peaks in the 2D [^1^H,^15^N] HSQC spectrum of MqsR (free *versus* MqsA_1-76_-bound MqsR). However, upon reaching a 1:1 M ratio, the 2D [^1^H,^15^N] HSQC spectrum of MqsA_1-76_-saturated MqsR showed the expected number of peaks (Fig. S7*B*). Due to the significant spectral changes, it was necessary to perform a sequence-specific backbone assignment of labeled MqsR bound to unlabeled MqsA_1-76_ (Fig. S7*C*). As expected, these observed CSPs align with previously reported crystal structures of the MqsR:MqsA complex (21) (Fig. S7, *D* and *E*).

**Figure 5:**
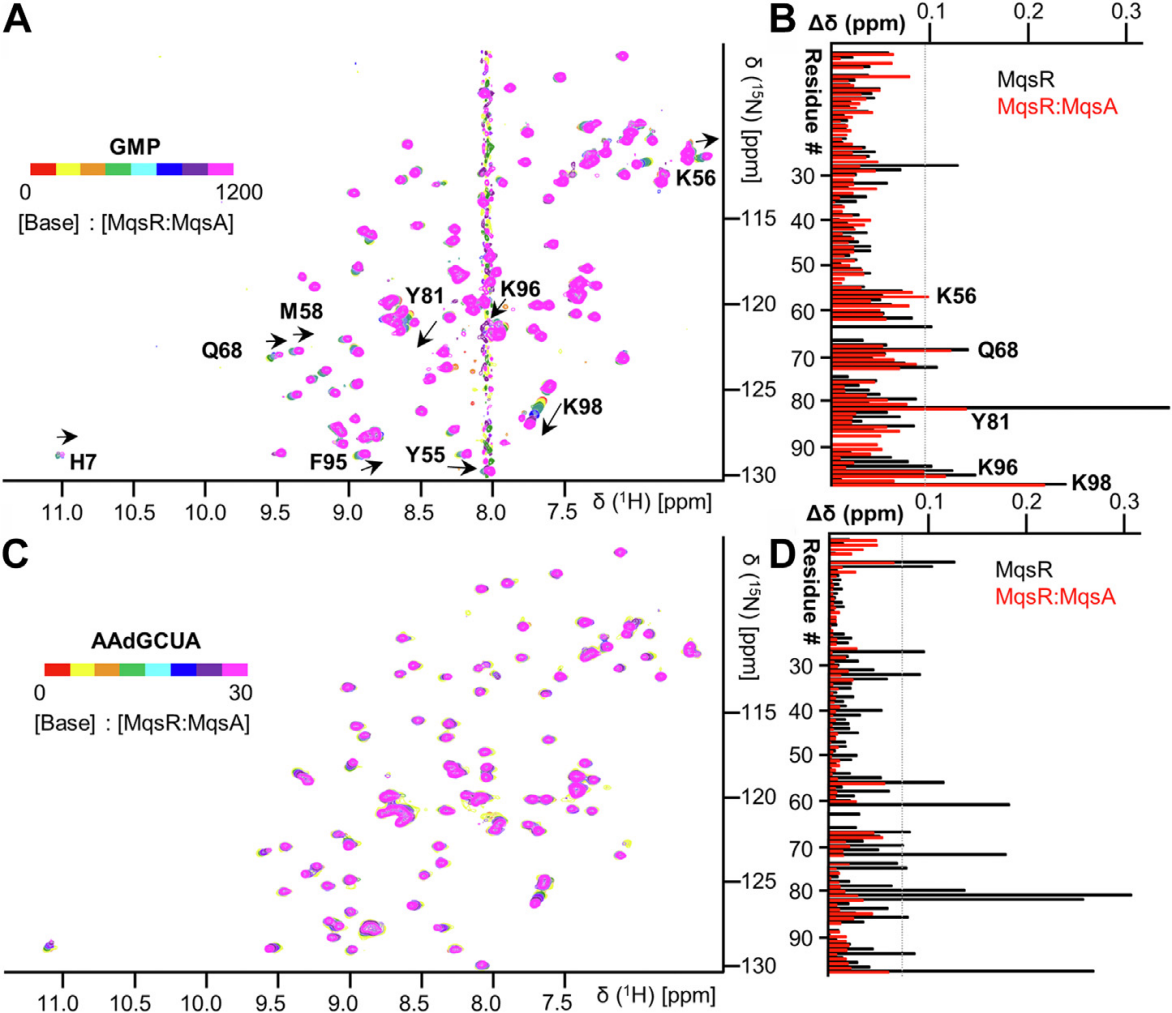
**MqsA sterically hinders substrate binding to MqsR orthogonal to the active site.***A*, NMR titrations of GMP to ^15^N-labeled MqsR incubated with an excess of MqsA_1-76_. *B*, associated CSP values; ^15^N-MqsR CSPs in *black* and ^15^N-MqsR:MqsA_1-76_ in *red*. *C* and *D*, the same as in Fig. 5, *A* and *B* but titrated with 5′-AAdGCUA-3′ substrate. CSP, chemical shift perturbation.

To define how MqsA inhibits MqsR activity, we next titrated distinct classes of substrates (GMP and 5′-AAdGCUA-3′) into the ^15^N-MqsR:MqsA_1-76_ complex. Titration of GMP into the ^15^N-MqsR:MqsA_1-76_ complex resulted in CSPs to multiple MqsR residues in the active site. Notably, both Tyr81 and Lys56, which we identified as the catalytic residues of MqsR, continue to exhibit large CSPs along with Gln68, Lys96, and Lys98; other residues that are distal to the active site, most noticeably Ser27, Tyr61, and Asp63, lose their ability to interact with substrate (Fig. 5, *A* and *B*). This demonstrates that the MqsR active site remains accessible and binds GMP. We then performed NMR titrations with the noncleavable 5′-AAdGCUA-3′ substrate. In contrast to both the number and extent of the CSPs observed in titrations with free MqsR, no CSPs ≥ 1σ_0_ were observed in titrations with the MqsR:MqsA_1-76_ complex (Fig. 5, *C* and *D*). That is, mRNA substrates are no longer able to bind MqsR when MqsR is bound to its cognate antitoxin. As a consequence, mRNA substrates are not cleaved. This shows that, despite the presence of a fully accessible MqsR active site, MqsA inhibits MqsR activity by sterically inhibiting substrate binding.

## Discussion

Type II TA pairs play critical roles in regulating the bacterial stress response. One established type II TA systems is the MqsR:MqsA system. Indeed, while early structural and functional studies of MqsR showed it was an RNase (20, 21, 27), we still lacked detailed molecular information describing how MqsR mediates cleavage, how MqsR recruits its substrates, if MqsR exhibits substrate specificity preferences beyond the main recognition sequence, and how MqsA inhibits MqsR. To answer these questions, we studied the MqsR:MqsA system using biomolecular NMR CSP experiments and mRNA cleavage assays, which revealed that MqsR catalysis, substrate binding, and MqsA-mediated inhibition is highly atypical compared to canonical ribosome-independent RNases and other toxins.

First, while many ribosome-independent RNases use a canonical glutamate/histidine pair to mediate mRNA cleavage (Fig. S1), our data showed that neither a glutamate nor histidine is critical for MqsR activity. Instead, we identified that two comparatively atypical residues, a tyrosine (Tyr81) and a lysine (Lys56), that are essential for mRNA binding and hydrolysis. The evidence supporting this conclusion is that both residues, when mutated to an alanine, completely block mRNA cleavage (Fig. 4). This conclusion is further supported by the observation that the positions of both Tyr81 and Lys56 structurally overlap with the experimentally confirmed catalytic residues from other ribosome-independent and ribosome-dependent RNases (Fig. S1). Finally, Tyr81 is one of the most highly conserved residues in MqsR (Fig. S8). These data demonstrate that, for MqsR, the general acid-base chemistry used for RNase phosphodiester bond cleavage is most likely performed by Tyr81 and Lys56. Either residue, in the deprotonated state, could function as the catalytic base. Although the pKa’s for both residues are high (pKa: 10.2, tyrosine; 10.5 lysine), their deprotonated states would be easily stabilized by the high number of positively charged residues adjacent to both residues (Arg72, Arg74, Lys96, and Lys98), facilitating their ability to function as a nucleophile. These same positively charged residues also likely stabilize the negatively charged 2,3-cyclic phosphate transition state. While neither a tyrosine nor a lysine are canonical nucleophiles, both amino acids have been shown to function as catalytic bases in multiple enzymes, including Lys52 of the RelE RNase (38), Lys25/Arg33/Lys60 of Colicin E5 (26), Tyr324 of Sialidase (39), and Lys157/Tyr150/Arg252 of the CdiA toxin domain from *Klebsiella pneumonia* (CdiA-CT^Kp342^) (40). The latter toxin domain, like MqsR, also use a Tyr/Lys pair for mRNA cleavage; despite the observation that it is identified by the DALI server to be one of the toxins most similar to MqsR, an overlay of the two proteins shows that the active site locations differ (Fig. S9), with that of CdiA-CT^Kp342^ being more similar to colicin D (40) and MqsR more similar to that of RNase SA and YoeB (Fig. S1).

**Figure 6:**
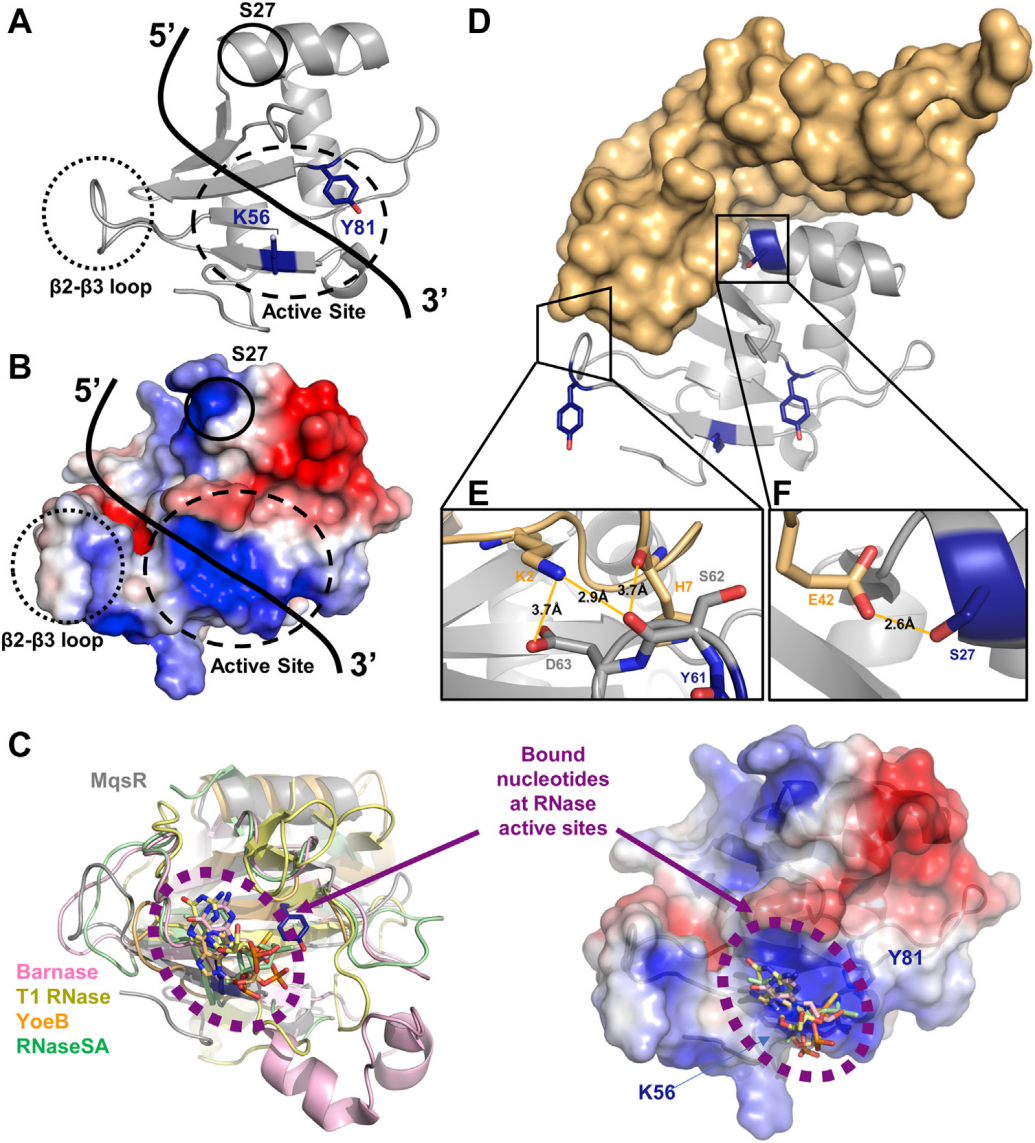
**The mechanism of action and inhibition of MqsR.***A*, the structure of MqsR with the two catalytic Lys56 and Tyr81 residues in *dark blue*. The active site, the β2-β3 loop, and Ser27 have been *circled*. *B*, the electrostatic potential map of MqsR in the same orientation as (*A*). *C*, *left*, overlay of MqsR (*gray*) with RNases crystallized with mRNA substrates/substrate analogs [Barnase (PDBID 1BRN (36), *pink*), T1 RNase (PDBID 1GSP (46); *yellow*), YoeB (PDBID 6OXA (16), *orange*), and RNase SA (PDBID 1RSN (47), *green*)] with nucleotides bound at the catalytic site shown as *sticks*. *Right*, same view but shown with MqsR electrostatic potential map shown as a surface. *D*, the MqsR:MqsA structure. MqsA is shown as a surface in *light orange* and MqsR is shown in *gray* as a *cartoon*. *E* and *F*, MqsA interacts with key residues distal to the active site of MqsR, preventing substrate binding and inactivating MqsR. Nitrogen is colored *red* and Oxygen is colored *blue*.

Second, while MqsR, like many ribosome-independent RNases, is guanosine directed with a short recognition sequence motif (*i.e.*, MqsR is 5′-GC-3′), our CSP studies demonstrate that MqsR also exhibits preferences outside this motif. Specifically, MqsR prefers purines rather than pyrimidines (A > G >>> U > C) in the −2 and −1 nucleotide positions relative to the consensus GC sequence and substrates with these sequences bind MqsR with higher affinities (Table 1). *In vitro* cleavage assays of MqsR with various RNA substrates (Fig. 1*A* and Table S1) also show that MqsR preferentially targets GCU sites that are flanked by adenines at the −2 and −1 position. NMR CSP experiments also identified the MqsR residues outside the active site that mediate binding to mRNA substrates: the β2-β3 loop (residues 61–67) and Ser27 (Fig. 4). In particular, the residues in the β2-β3 loop exhibit intermediate timescale (μs/ms) dynamics and also adopt different conformations in the MqsR:MqsA crystal structure (21), indicating flexibility to accommodate substrate. Taken together, these data show that mRNA substrates bind an extended channel along the front MqsR protein (Fig. 6*A*), with the −2 and −1 nucleotides likely binding the cleft between Ser27 and the β2-β3 loop and the 5′-GC-3′ motif positioned for nucleophilic attack at the active site pocket. Consistent with this, the MqsR electrostatic surface potential shows that this cleft is highly positively charged, as would be expected for a protein that coordinates the negatively charged backbone of mRNA substrates (Fig. 6*B*). Furthermore, the active site pockets of other RNases whose structures have been determined bound to nucleotide substrates also overlap at this position (Fig. 6*C*).

Third, the discovery of the importance of the MqsR β2-β3 loop also provided the first hints into the atypical mechanism used by MqsA to inhibit MqsR; namely, that MqsA, which also binds this loop, does *not* bind or distort the MqsR active site (*i.e.*, like RelB when bound to RelE (24)) but instead simply inhibits mRNA binding. To test this, we repeated the CSP experiments but titrated the substrates (GMP, mRNAs) with the MqsR:MqsA complex, that is, when MqsR is bound to its cognate antitoxin. The CSP data confirmed that the active site is fully accessible in the complex, as titrations with GMP resulted in CSPs nearly identical to those observed with MqsR alone (Fig. 5, *A* and *B*). However, a completely different result was obtained when titrations were performed with mRNA substrates. In contrast to the multiple, significant CSPs observed when *free* MqsR was titrated with mRNA substrates (Figs. 2, 3, S2–S4), almost no significant CSPs were observed for the titrations with *MqsA-bound* MqsR (Fig. 5, *C* and *D*). Thus, MqsA inhibits MqsR activity not by occluding or altering the active site but instead simply prevents mRNA substrates binding MqsR. Analysis of the MqsR:MqsA crystal structure (Fig. 6*D*) reveals how this is achieved at a molecular level. Although the MqsR residues that constitute the active site are fully accessible in the MqsR:MqsA complex, consistent with their ability to bind mononucleotides, the residues that engage the −2 and −1 nucleotides in mRNA substrates, Ser27 and the β2-β3 loop, interact extensively with MqsA (21). In particular, both regions in MqsR form multiple hydrogen bonds with MqsR (Glu42_MqsA_-Ser27_MqsR_; Lys2_MqsA_-Asp63_MqsR_, Lys2_MqsA_-Ser62_MqsR_, His7_MqsA_-Asp62_MqsR_) (Fig. 6, *E* and *F*). Thus, while in free MqsR, the MqsR β2-β3 loop residues are flexible and primed to cradle the substrate, in the MqsR:MqsA complex, they make extensive interactions with multiple MqsA residues, preventing this loop from binding mRNA substrates.

In conclusion, these data provide the most complete view of the unique mechanisms used by MqsR to bind and hydrolyze mRNA and how MqsA binding potently inhibits these activities. In particular, to our knowledge, MqsR is the only RNase that mediates mRNA cleavage using a Tyr/Lys catalytic pair (41), with its cognate antitoxin, MqsA, inhibiting MqsR activity by simply preventing substrate binding. Further studies into both this unique active site and the novel new inhibitor loop may reveal how MqsR recognizes the specificity of the distal nucleotides and may lead to drugs that can selectively inhibit RNases or even prevent certain RNA strands from being cleaved while retaining general RNase activity.

## Experimental procedures

### Protein purification

Protein expression and purification was performed largely as described previously (21). Briefly, BL21(DE3) cells (Invitrogen) were cotransformed with pET30a-MqsR (untagged) and pCA21a-MqsA_1-76_ (N-terminal His_6_ tag) plasmids. Cells were grown at 37 °C in LB broth supplemented with appropriate antibiotics to an optical density (A_600_) of 0.6–0.8. IPTG was added to a final concentration of 0.5 mM and the cultures were incubated for an additional 4 h before being harvested *via* centrifugation. Pellets were stored at −80 °C.

Proteins were purified by suspending pellets in lysis buffer (500 mM NaCl, 50 mM Tris pH 8.0, 0.1% Triton X-100, 5 mM imidazole) containing EDTA-free protease inhibitor tablets (Roche) and lysed by high pressure cell homogenization (Avestin C3). Cell debris was removed by centrifugation at 42,000*g* for 45 min at 4 °C. The His_6_-MqsA/MqsR complex was incubated with nickel-NTA resin and washed with five column volumes of buffer A (500 mM NaCl, 50 mM Tris pH 8.0, 5 mM imidazole). Unfolded MqsR was eluted using buffer A supplemented with 6 M guanidine hydrochloride (Gdn-HCl). MqsR was refolded using stepwise dialysis in buffers containing decreasing amounts of Gdn-HCl followed by a final preparative gel filtration step (Superdex 75 16/60; GE Healthcare), following previously described methods (19). Uniformly, ^15^N- and ^15^N/^13^C-labeled MqsR were produced using the same procedures as above, except that the cells were grown in M9 minimal medium supplemented with [^15^N] ammonium chloride (1 g/l) and/or D-[^13^C] glucose (4 g/l).

The purification of His_6_-tagged MqsR protein used in activity assays was performed identically except that BL21(DE3) cells were transformed with plasmids pET28a-His_6_-MqsR (WT or mutant) and untagged pCA21a-MqsA_1-76_. Ni-NTA columns were washed with buffer A containing 6 M Gdn-HCl, and His_6_-MqsR was eluted by washing the columns with denaturing buffer B (500 mM NaCl, 50 mM Tris pH 8.0, 500 mM imidazole, 6 M Gdn-HCl). MqsR was refolded using stepwise dialysis in buffer A containing decreasing amounts of Gdn-HCl followed by a final preparative gel filtration step (Superdex 75 16/60; GE Healthcare). MqsR:MqsA_1-76_ complex was purified as above only without the use of Gdn-HCl. Untagged MqsA_1-76_ was purified as previously described (42).

### NMR spectroscopy

All NMR spectra were recorded at 298 K on either a Bruker Avance 500 MHz or a Bruker Avance IIHD 850 MHz spectrometer, both equipped with a TCI HCN z-gradient cryoprobe. Sequence-specific backbone resonance assignments for free and MqsA-bound MqsR were obtained by analyzing 2D [^1^H,^15^N] HSQC spectrum, 3D HNCA, 3D HN(CO)CA, 3D HNCACB, and 3D CBCA(CO)NH spectra. Data were processed using TopSpin (Bruker) and assignments were carried out using CARA (http://cara.nmr.ch/).

For NMR-based titration studies, ^15^N-labeled MqsR was diluted to 50 μM into NMR Buffer (50 mM NaCl, 20 mM Na phosphate pH 6.5, 0.5 mM TCEP) with 10% v/v D_2_O. Ligands were dissolved in NMR buffer and added stepwise to ^15^N-labeled MqsR. Data were analyzed using CCPNmr Analysis (26), SigmaPlot v.12.5 (Systat Software), and Excel’s Solver Add-on. Backbone amide CSPs were calculated using the formula: $CSP=\sqrt{\Delta \delta H^{2}+{\frac{\Delta \delta N}{10}}^{2}}$, where ΔδH and ΔδN are the total chemical shifts experienced in the proton and nitrogen dimensions. Residue-specific and global K_D_ (dissociation constant) values were calculated following a protocol described in (32). Residue-specific K_D_ values were calculated by fitting to the equation: $CSP_{obs}=\frac{CSP_{\max}}{2\left[P\right]}\left(\left(\left[P\right]+\left[S\right]+K_{D}\right)-\sqrt{{\left(\left[P\right]+\left[S\right]+K_{D}\right)}^{2}-4\left[S\right]\left[P\right]}\right)$, where P and S are the concentration of ^15^N-labeled MqsR and substrate, respectively, CSP_obs_ is the experimentally obtained chemical shift value, and CSP_max_ is the largest experimentally obtained chemical shift of the residue. A global K_D_ was determined by averaging the residue-specific K_D_’s of all residues with a CSP ≥ 1σ_0_ (33). For titrations which did not reach complete substrate saturation, the reported K_D_ values will be an overestimation.

### Differential scanning fluorimetry

His_6_-MqsR (WT and mutants) were mixed 1:1 with 2× SYPRO Orange (Invitrogen) in Reaction Buffer (100 mM NaCl, 10 mM Tris pH 7.5, 0.5 mM TCEP) and fluorescence was assayed in a BioRad CFX Connect rt-PCR instrument over a thermal melt curve from 4 to 80 °C. Melting temperature (T_m_) was determined by calculating the minima of the first derivative of each reaction in CFX Maestro software (BioRad).

### MqsR activity assay

mRNAs were synthesized from DNA oligos (IDT). DNA was reverse transcribed (Ampliscribe T7-Flash Transcription Kit, Lucigen) and the resulting mRNA was purified by successive extractions first using a phenol:chloroform:isoamyl alcohol (25:24:1) mixture and then with multiple 100% chloroform extractions until all contaminants were removed. RNA was then precipitated in 100% ice cold ethanol. The ethanol was removed and the sample air dried then resuspended in reaction buffer.

His_6_-MqsR (30 ng) was incubated with 1 μg of RNA at 25 °C or 37 °C for 3 or 5 h, respectively. Activity was inhibited by incubating samples at 65 °C for 5 min to stop the reaction and mixed 1:1 with a 2× Invitrogen sample loading dye. Samples were subjected to 15% TBE-Urea gel analysis (Life Technology) and visualized by incubation with a 0.1% SYBRsafe solution. Gels were imaged using a BioRad ChemiDOC MP imaging system.

### Statistical analysis

Differential scanning fluorimetry assays were performed in replicates of 6. MqsR activity assays were performed in biological replicates of 3. Each biological replicate was technically replicated three times, yielding a total of nine replicates for quantification. The activity of MqsR (wt and mutants) was determined using intensity calculations from ImageLab software (BioRad). Cleavage extent was determined by calculating the sum of the intensities of the two primary cleaved RNA bands and normalizing these intensities to complete intensity of the lane, a procedure equivalent to total protein normalization in Western blot quantification (43, 44, 45).

When comparing activity assays, an ANOVA test using Dunnett’s multiple comparisons procedure was used. Analysis was performed in SigmaPlot v.12.5 (Systat Software). ∗, ∗∗, and ∗∗∗ denotes *p* < 0.05, *p* < 0.01, and *p* < 0.001, respectively.

## Data availability

All NMR chemical shifts have been deposited in the BioMagResBank (BMRB 51502, 51503). Original MqsR activity assay gel image files are available upon request.

## Supporting information

Supporting information are available online.

## Conflict of interest

The authors declare no competing interests. The funders had no role in study design, data collection and analysis, decision to publish, or preparation of the article.
