# Supplementary material for: MqsR is a noncanonical microbial RNase toxin that is inhibited by antitoxin MqsA via steric blockage of substrate binding
Source: J Biol Chem. 2022 Sep 24;298(11):102535. doi: 10.1016/j.jbc.2022.102535 (PMC9636575; doi:10.1016/j.jbc.2022.102535)
Supplement: Supplemental Tables S1–S5 and Fig. S1–S9 [file mmc1.pdf]

Supplemental Information:

MqsR is a noncanonical microbial RNase toxin that is inhibited by antitoxin MqsA via steric blockage of substrate binding

Victor Yu[1], Erik Ronzone[2], Dana Lord[2], Wolfgang Peti[3] and Rebecca Page[1,*]

[1]Department of Cell Biology, University of Connecticut Health Center, Farmington, Connecticut, 06030, USA; [2]Department of Molecular Biology, Cell Biology and Biochemistry, Brown University, Providence, RI, 02919, USA; [3]Department of Molecular Biology and Biophysics, University of Connecticut Health Center, Farmington, Connecticut, 06030, USA

Contents:

(1) **Table S1.** MqsR substrates.
(2) **Table S2.** Residues use to calculate global K_D for mononucleotide substrates.
(3) **Table S3.** Residues use to calculate global K_D for non-hydrolyzable mRNA substrates.
(4) **Table S4**. Residues that exhibited significant CSPs in the mononucleotide and non-hydrolyzable mRNA substrate titrations (11 distinct substrates total).
(5) **Table S5**. MqsR constructs and their melting temperature.
(6) **Figure S1.** Active sites of RNases homologous to MqsR bound to a substrate mimic.
(7) **Figure S2**. MqsR-UMP NMR Titrations.
(8) **Figure S3.** -2 Nucleotide NMR Titrations.
(9) **Figure S4.** -1 Nucleotide NMR Titrations.
(10) **Figure S5.** MqsR wt and variant mRNA cleavage assays at 37 °C.
(11) **Figure S6.** MqsR wt and variant mRNA cleavage assays at 25 °C.
(12) **Figure S7.** MqsA binds distally to the MqsR active site.
(13) **Figure S8.** The active site of MqsR is the most well conserved region.
(14) **Figure S9.** Overlay of MqsR and CdiA-CT^{Kp342}

Table S1: MqsR substrates. Substrates used for activity assays. GCU sites highlighted in yellow and GC sites highlighted in green.

Substrate	Sequence
**wt MqsA RNA**	AGAAGGAGAUAUACAUAUGAAAUGUCCGGUUUGCCACCAGGGAGAAAUGGUUUCUGGCAUUAAAGAUAUUCCA<br>UACACCUUCCGUGGACGAAAAACAGUAUUGAAAGGUAUCCACGGUUUAUAUUGUGUCCAUUGCGAAGAGAGCA<br>UCAUGAAUAAAGAAGAGUCAGAUGCUUUCAUGGCGCAAGUAAAGGCAUUUCGGGCUUCGGUGAAUGCCGAAAC<br>AGUGGCACCUGAAUUUAUAGUGAAGGUUCGAAAAAAGCUCUCUCUUACCCAAAAAGAGGCAAGCGAAAUUUUU<br>GGGGGAGGUGUAAAUGCGUUUUCGCGUUACGAAAAAGGCAAUGCCCAACCUCAUCCUUCCACAAUCAAACUUU<br>UACGUGUUCUGGAUAAGCAUCCAGAACUAUUGAAUGAAAUCCGUUAA
**NoGCU MqsA RNA**	AGAAGGAGAUAUACAUAUGAAAUGUCCGGUUUGCCACCAGGGAGAAAUGGUUUCUGGCCUUAAAGAUAUUCCA<br>UACACCUUCCGUGGACGAAAAACAGUAUUGAAAGGUAUCCACGGUUUAUAUUGUGUCCAUUGCGAAGAGAACA<br>UCAUGAAUAAAGAAGAGUCAGAUGUUUUCAUGGCCCAAGUAAAGGUCUUUCGGGCCUCGGUGAAUGCCGAAAC<br>AGUGACACCUGAAUUUAUAGUGAAGGUUCGAAAAAAGCCCUCUCUUACCCAAAAAGAGGUUAGCGAAAUUUUU<br>GGGGGAGGUGUAAAUGCGUUUUCGCGUUACGAAAAAGGGAAUGCCCAACCUCAUCCUUCCACAAUCAAACUUU<br>UACGUGUUCUGGAUAAUCAUCCAGAACUAUUGAAUGAAAUCCGUUAA
**NoGC MqsA RNA**	AGAAGGAGAUAUACAUAUGAAAUGUCCGGUUUUCCACCAGGGAGAAAUGGUUUCUGGUCUUAAAGAUAUUCCA<br>UACACCUUCCGUGGACGAAAAACAGUAUUGAAAGGUAUCCACGGUUUAUAUUGUGUCCAUUGTGAAGAGAACA<br>UCAUGAAUAAAGAAGAGUCAGAUGUUUUCAUGGACCAAGUAAAGGTCUUUCGGGACUCGGUGAAUCCCGAAAC<br>AGUGACACCUGAAUUUAUAGUGAAGGUUCGAAAAAAUCCCUCUCUUACCCAAAAAGAGGUUAGGGAAAUUUUU<br>GGGGGAGGUGUAAAUCCGUUUUCGGGUUACGAAAAAGGGAAUUCCCAACCUCAUCCUUCCACAAUCAAACUUU<br>UACGUGUUCUGGAUAAUCAUCCAGAACUAUUGAAUGAAAUCCGUUAA
**GC+1GCU MqsA RNA**	AGAAGGAGAUAUACAUAUGAAAUGUCCGGUUUGCCACCAGGGAGAAAUGGUUUCUGGCCUUAAAGAUAUUCCA<br>UACACCUUCCGUGGACGAAAAACAGUAUUGAAAGGUAUCCACGGUUUAUAUUGUGUCCAUUGCGAAGAGAACA<br>UCAUGAAUAAAGAAGAGUCAGAUGUUUUCAUGGCCCAAGUAAAGGUCUUUCGGGCCUCGGUGAAUGCCGAAAC<br>AGUGACACCUGAAUUUAUAGUGAAGGUUCGAAAAAAGCUCUCUCUUACCCAAAAAGAGGUUAGCGAAAUUUUU<br>GGGGGAGGUGUAAAUGCGUUUUCGCGUUACGAAAAAGGGAAUGCCCAACCUCAUCCUUCCACAAUCAAACUUU<br>UACGUGUUCUGGAUAAUCAUCCAGAACUAUUGAAUGAAAUCCGUUAA
**1GCU MqsA RNA**	AGAAGGAGAUAUACAUAUGAAAUGUCCGGUUUUCCACCAGGGAGAAAUGGUUUCUGGUCUUAAAGAUAUUCCA<br>UACACCUUCCGUGGACGAAAAACAGUAUUGAAAGGUAUCCACGGUUUAUAUUGUGUCCAUUGTGAAGAGAACA<br>UCAUGAAUAAAGAAGAGUCAGAUGUUUUCAUGGACCAAGUAAAGGTCUUUCGGGACUCGGUGAAUCCCGAAAC<br>AGUGACACCUGAAUUUAUAGUGAAGGUUCGAAAAAAGCUCUCUCUUACCCAAAAAGAGGUUAGGGAAAUUUUU<br>GGGGGAGGUGUAAAUCCGUUUUCGGGUUACGAAAAAGGGAAUUCCCAACCUCAUCCUUCCACAAUCAAACUUU<br>UACGUGUUCUGGAUAAUCAUCCAGAACUAUUGAAUGAAAUCCGUUAA

Table S2: Residues use to calculate global K_D for mononucleotide substrates.

Substrate	MqsR residue*																
	H7	S27	K56	M58	Y61	D63	W67	Q68	R72	Q79	Y81	I84	T85	V93	S94	F95	K96	K98
GMP		X			X	X		X	X	X	X		X	X	X	X	X	X
UMP	X	X	X	X	X		X				X	X				X		X

*Residues with a CSP ≥1σ_0 in each titration were used to calculate their binding affinity.

Table S3: Residues use to calculate global K_D for non-hydrolyzable mRNA substrates.

Substrate	MqsR residue*																					
	**H7**	**T8**	**S27**	**A32**	**K56**	**Y61**	**D63**	**I66**	**W67**	**V70**	**R72**	**R74**	**L75**	**V80**	**Y81**	**L82**	**T85**	**V86**	**S94**	**K96**	**E97**	**K98**
**AdGCUA**	X		X		X	X		X			X				X	X	X	X	X	X	X	X
**AAdGCUA**	X	X	X	X	X	X			X	X	X		X	X	X	X		X	X			X
**AAAdGCUA**	X	X	X	X	X	X			X		X	X	X	X	X			X	X			X
**GAdGCUA**	X	X	X	X	X	X			X		X			X	X	X		X	X			X
**UAdGCUA**	X	X	X	X	X	X			X		X		X	X	X	X			X	X		X
**CAdGCUA**	X	X	X	X	X	X			X		X		X	X	X	X		X	X			X
**AGdGCUA**	X	X	X	X	X	X	X		X		X			X	X	X			X	X		X
**AUdGCUA**	X	X	X	X	X	X			X	X	X		X	X	X	X			X	X		X
**ACdGCUA**	X	X	X	X	X	X					X		X	X	X	X			X	X		X

*Residues with a CSP $\geq 1\sigma_0$ in each titration were used to calculate their binding affinity.

Table S4: Residues that exhibited significant CSPs (≥1σ_0) in the mononucleotide and non-hydrolyzable mRNA substrate titrations (11 distinct substrates total).

Residue	Number of substrates that the residue experienced a significant CSP			
	1σ_0	1.5σ_0	2σ_0	3σ_0
**H7**	10	9	9	7
**T8**	7	5	3	
**S27**	11	11	9	7
**A32**	8	4	2	
**K56**	10	10	8	2
**M58**	1	1		
**Y61**	11	10	9	9
**D63**	1	1		
**I66**	1	1	1	
**W67**	8	3	2	
**Q68**	1	1	1	1
**V70**	1			
**R72**	10	10	8	8
**R74**	1			
**L75**	7	2		
**Q79**	1			
**V80**	8	7	6	4
**Y81**	11	11	11	11
**L82**	8	8	8	8
**K83**	1			
**I84**	1			
**T85**	1	1	1	
**V86**	5			
**V93**	1			
**S94**	10	10	3	
**F95**	2	3	2	
**K96**	6	6	6	4
**E97**	1	1		
**K98**	11	11	11	11

Table S5: MqsR variant melting temperatures.

MqsR variant*	T_m (°C)	ΔT_m (°C)
**wt**	44.0 ± 0.2	N/A
**H7A**	36.4 ± 0.3	-7.6
**K56A**	38.5 ± 0.4	-5.5
**Y61A**	44.1 ± 0.2	0.1
**R72A**	38.4 ± 0.5	-5.6
**Y81A**	35.4 ± 0.1	-8.6
**L82I**	46.3 ± 0.1	2.3
**L82V**	42.3 ± 0.2	-1.7
**K98A**	44.6 ± 0.2	0.6

*n=6 for each variant

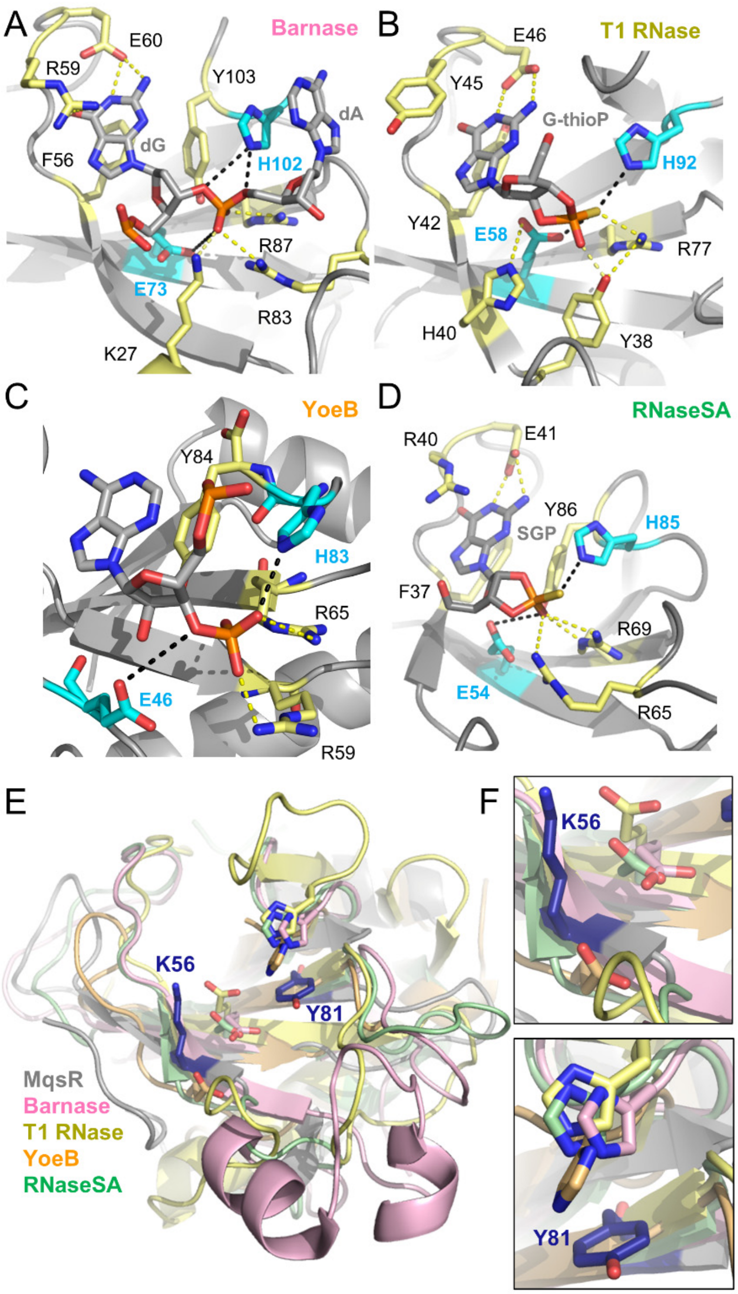

Figure S1: RNase active sites. *A-D.* Active sites of RNases homologous to MqsR (grey) bound to substrate mimics. The two catalytic residues (His and Glu) colored cyan. Other residues that mediate substrate binding highlighted in yellow. Oxygen colored red. Nitrogen, blue. Phosphorus, orange. A, Barnase (PDBID 1BRN, pink) B, T1 RNase (PDBID 1GSP, yellow), C, YoeB (PDBID 6OXA, orange), D, RNase SA (PDBID 1RSN, green). *E*. MqsR overlayed with homologous RNases, with active site residues shown as sticks and labeled**.** MqsR (gray) with Lys56 and Tyr81 in blue sticks overlaid with Barnase (pink), RNase T1 (yellow), YoeB (orange), RNase Sa (green). The catalytic Glu and His residues of the other RNases shown in sticks. *F.* Zoom in views of overlays in E, illustrating that Lys56 overlays closely with the catalytic Glu residues while Tyr81 is near the catalytic His residues.

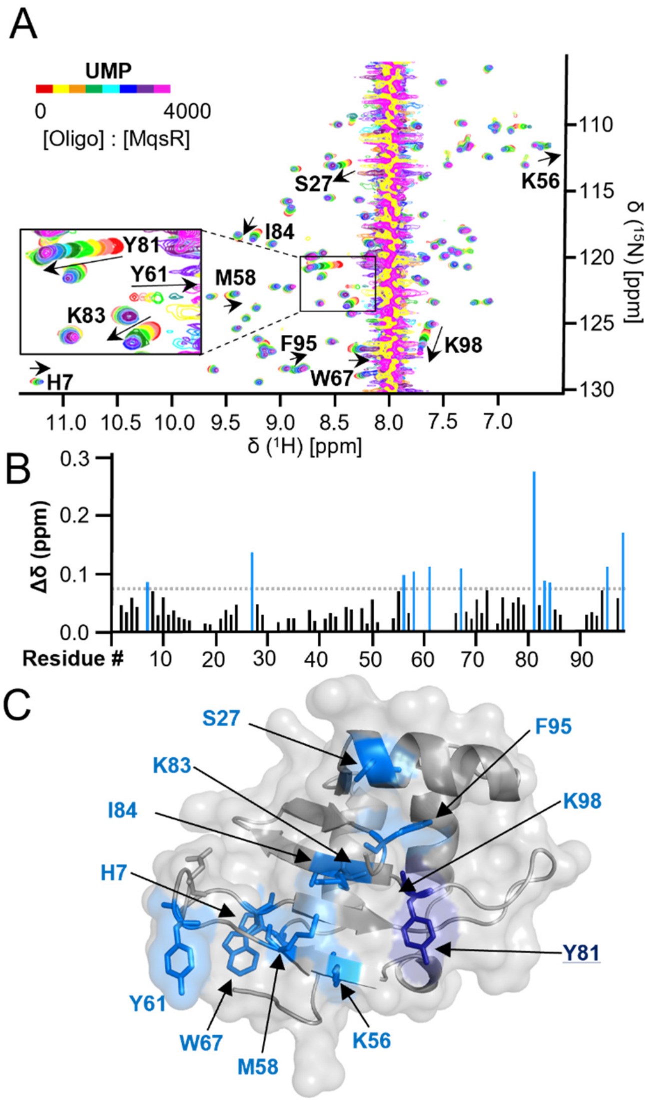

Figure S2: MqsR-UMP NMR Titrations. *A.* Overlay of the 2D [^{1}H,^{15}N] HSQC spectrum of ^{15}N-labeled MqsR titrated with increasing concentrations of uridine monophosphate (UMP). Peaks experiencing chemical shift perturbations (CSPs) due to increasing GMP are indicated by arrows and labeled. *B.* CSPs for MqsR:UMP plotted by residue number. Residues with significant CSPs (>1σ_0; indicated by a dotted line) shown in blue. *C,* MqsR crystal structure with residues that exhibit significant CSPs (>1σ_0) shown as sticks, colored blue and labeled; these residues were used to calculate a global binding K_D in Table 1. Y81, which exhibits the largest CSP, is shown in dark blue.

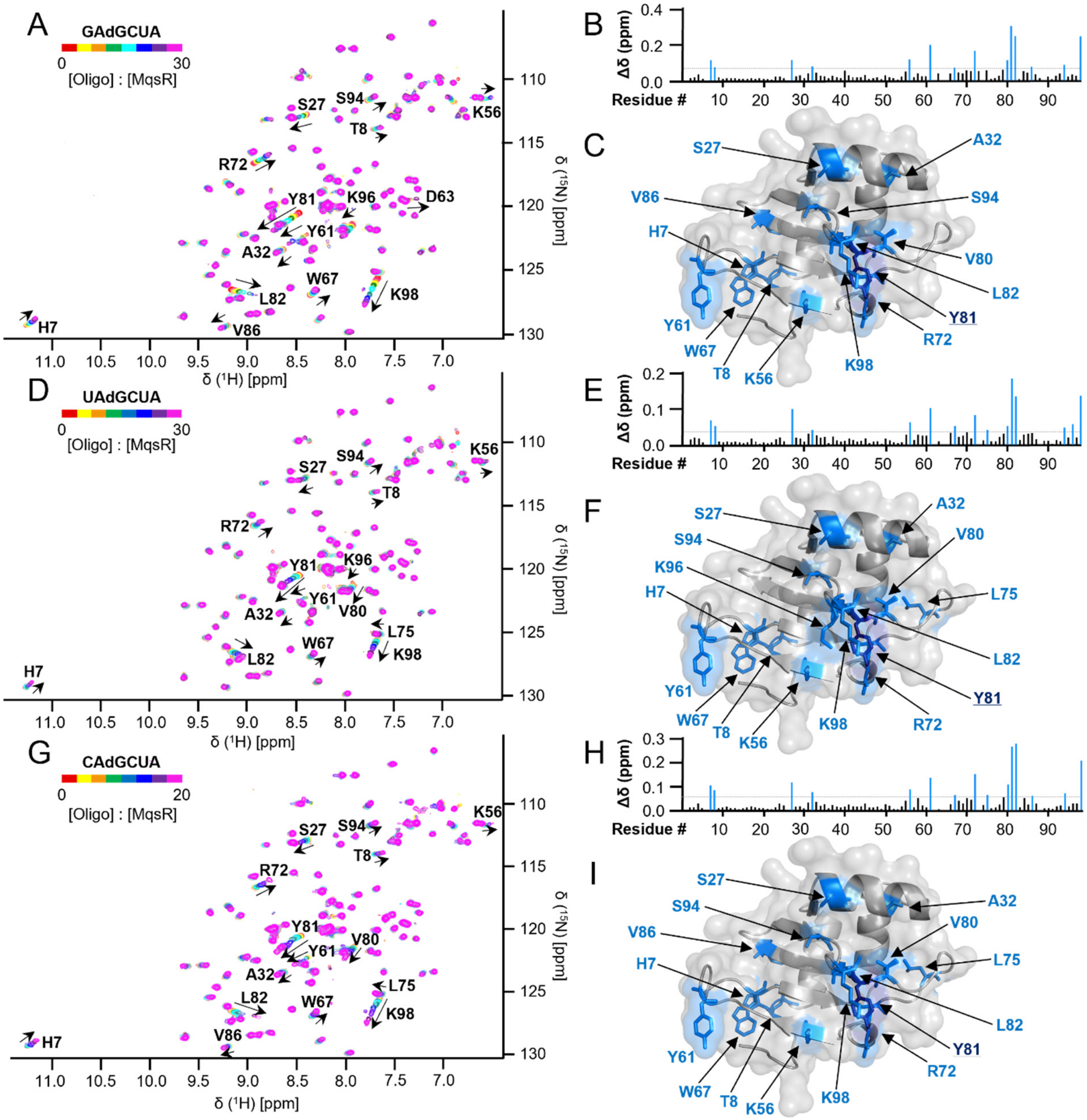

Figure S3: -2 Nucleotide NMR Titrations. *A.* Overlay of the 2D [^{1}H,^{15}N] HSQC spectra of ^{15}N-labeled MqsR with increasing concentrations of the non-hydrolyzable mRNA substrate 5'-GAdGCUA-3' (d indicates the base is deoxy; Oligo-GA). Peaks exhibiting CSPs due to the presence of the mRNA substrate are indicated by arrows and labeled. *B*. CSPs for MqsR:oligo-GA. Residues with significant CSPs (>1σ_0; indicated by a dotted line) are indicated in blue. *C.* MqsR crystal structure with residues that exhibit significant CSPs (>1σ_0) shown as sticks, colored blue and labeled; Y81, which exhibits the largest CSP, is shown in dark blue. *D*, *E*, *F*. Same as A, B and C, respectively, but with non-hydrolyzable mRNA substrate 5'-UAdGCUA-3' (Oligo-UA). G, H, I. Same as A, B and C, respectively, but with non-hydrolyzable mRNA substrate 5'-CAdGCUA-3' (Oligo-CA).

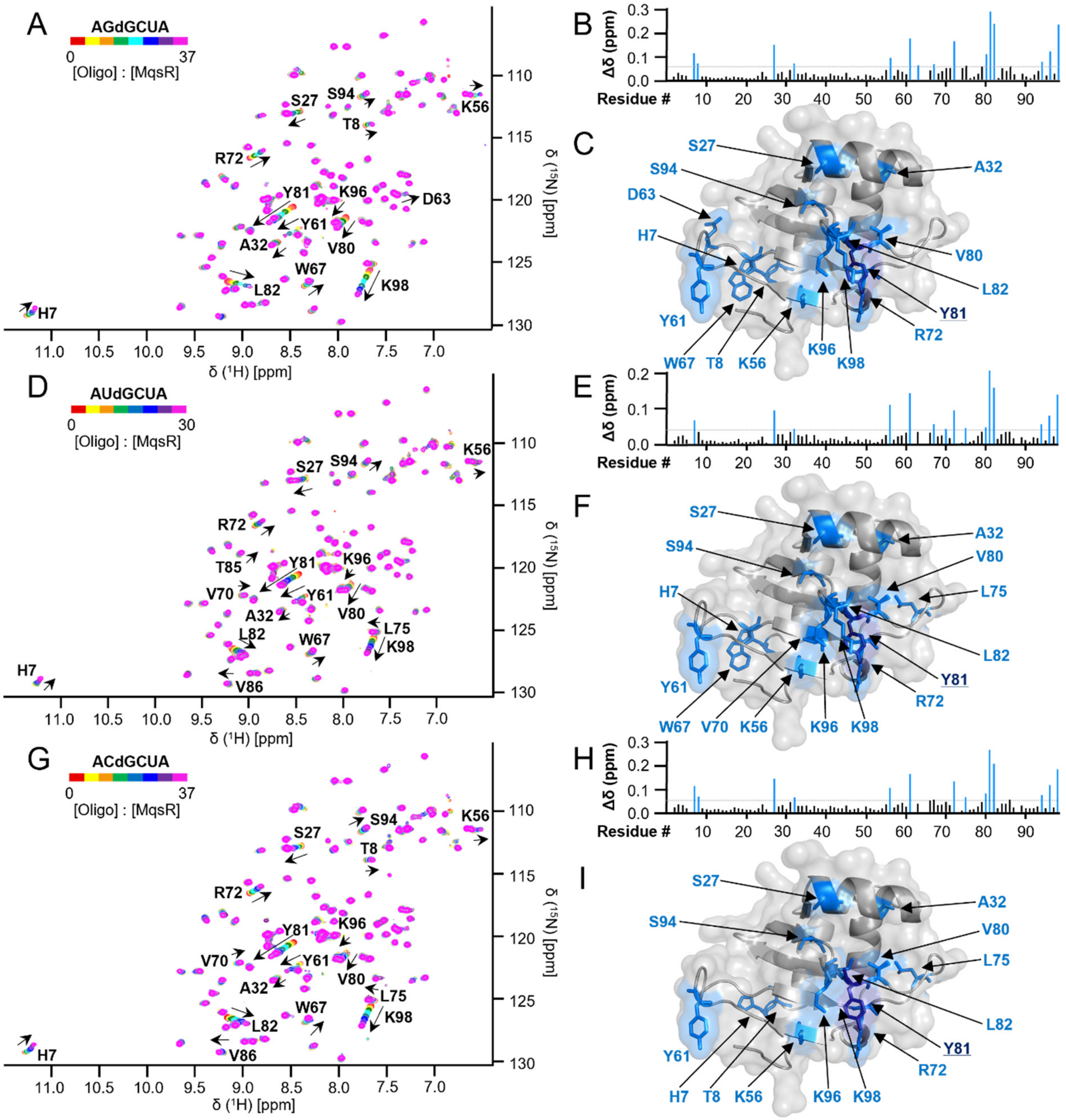

Figure S4: -1 Nucleotide NMR Titrations. *A.* Overlay of the 2D [^{1}H,^{15}N] HSQC spectra of ^{15}N-labeled MqsR with increasing concentrations of the non-hydrolyzable mRNA substrate 5'-AGdGCUA-3' (d indicates the base is deoxy; Oligo-AG). Peaks exhibiting CSPs due to the presence of the mRNA substrate are indicated by arrows and labeled. *B*. CSPs for MqsR:oligo-AG. Residues with significant CSPs (>1σ_0; indicated by a dotted line) are indicated in blue. *C.* MqsR crystal structure with residues that exhibit significant CSPs (>1σ_0) shown as sticks, colored blue and labeled; Y81, which exhibits the largest CSP, is shown in dark blue. *D*, *E*, *F*. Same as A, B and C, respectively, but with non-hydrolyzable mRNA substrate 5'-AUdGCUA-3' (Oligo-AU). G, H, I. Same as A, B and C, respectively, but with non-hydrolyzable mRNA substrate 5'-ACdGCUA-3' (Oligo-AC).

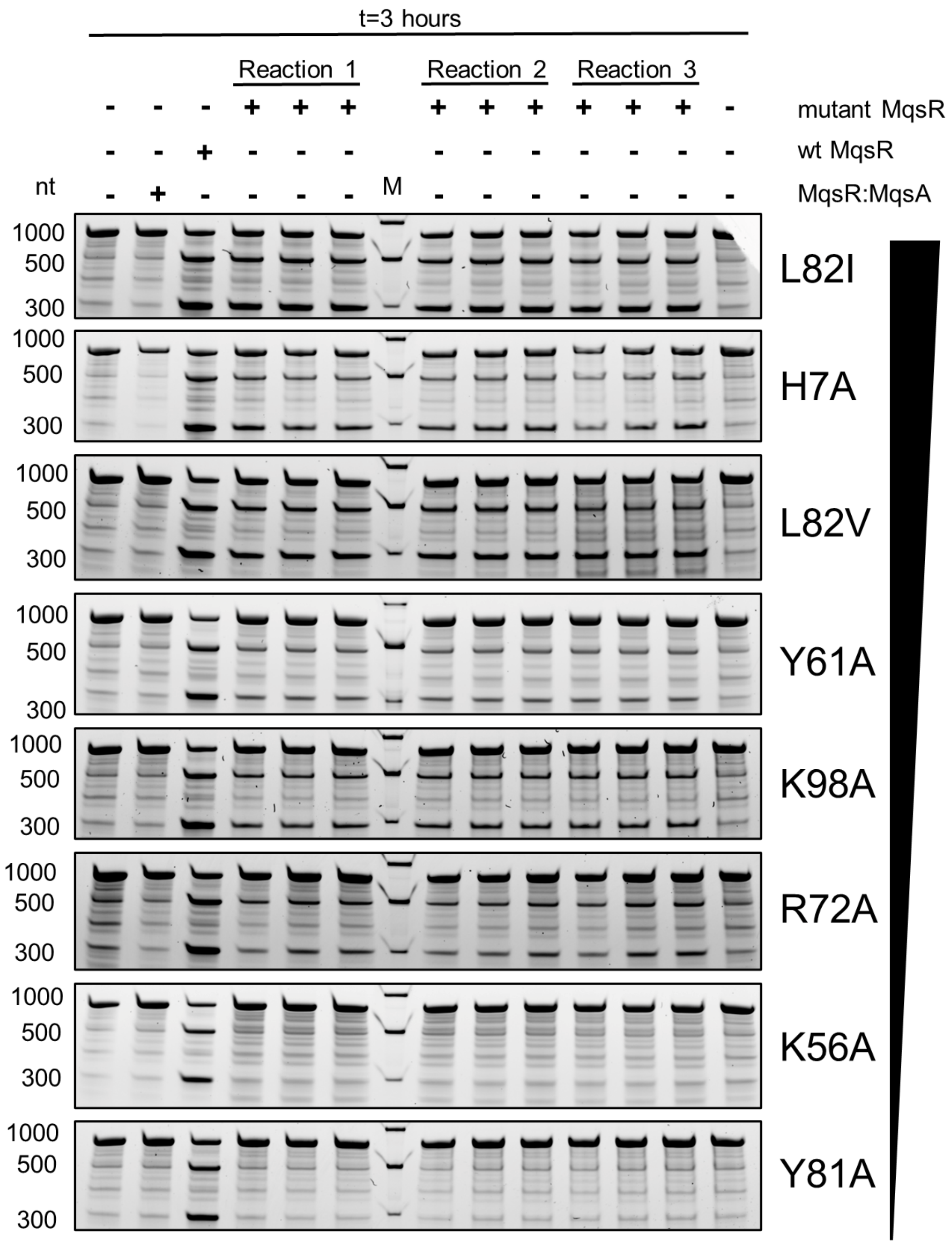

Figure S5: MqsR wt and variant mRNA cleavage assays at 37 °C. Cleavage assay data of MqsR mutants using a substrate with a singular preferred 5'-AAGCU-3' site (GC+1GCU MqsA RNA). Substrate sequence in Supplementary Table 1. Reactions were incubated at 37°C for 3 hours. All mutants assayed in three replicate reactions and each reaction ran in triplicate yielding an n=9 of each mutant. M, nt standards. The cleavage assay data for L82I and Y81A are also shown in Figure 4C in the main manuscript. Both panels are duplicated here to facilitate comparison between all MqsR variants.

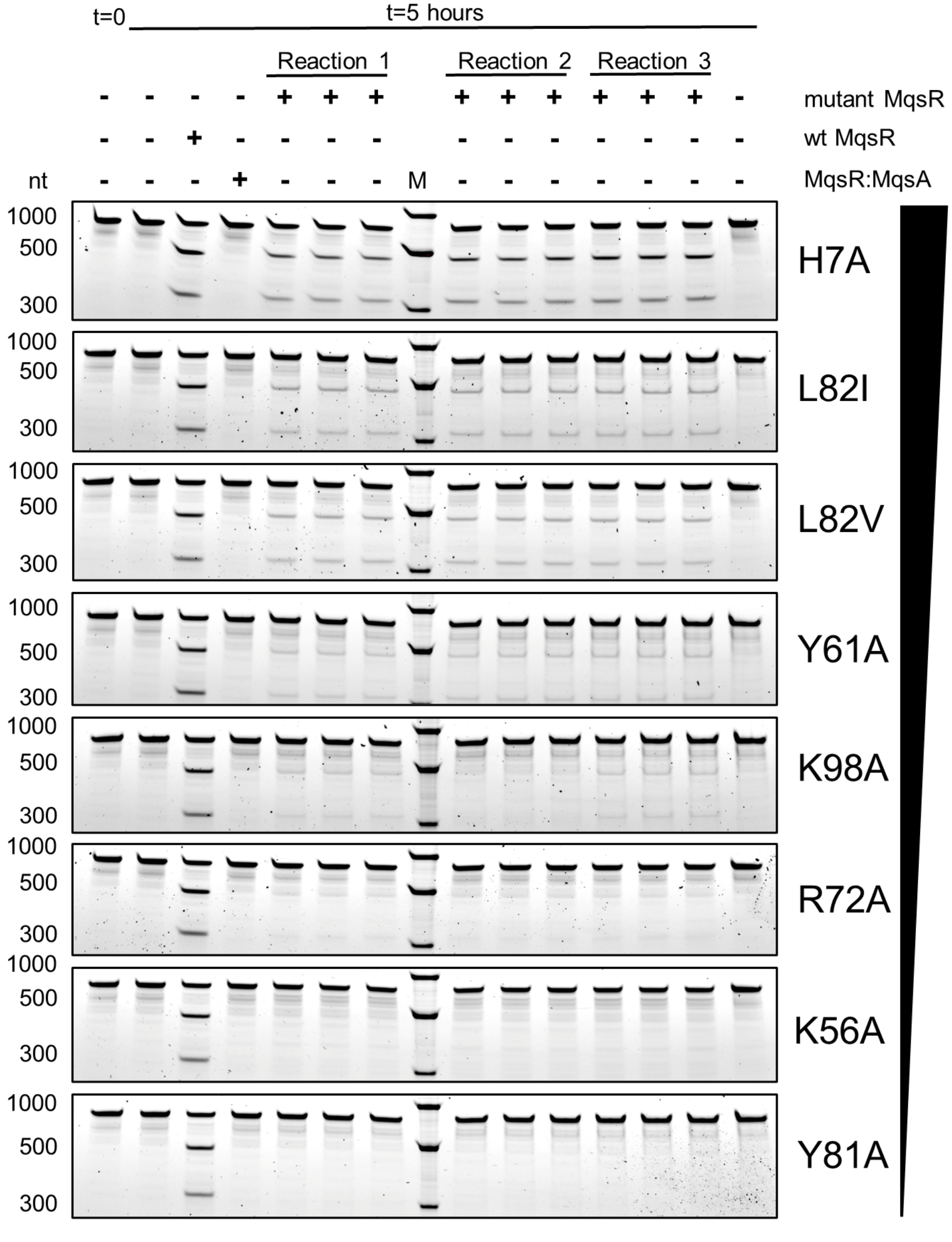

Figure S6: MqsR wt and variant mRNA cleavage assays at 25 °C. Cleavage assay data of MqsR mutants using a substrate with a singular preferred 5'-AAGCU-3' site (1GCU MqsA RNA). Substrate sequence in Supplementary Table 1. Reactions were incubated at 25°C for 5 hours. All mutants assayed in three replicate reactions and each reaction ran in triplicate yielding an n=9 of each mutant. M, nt standards.

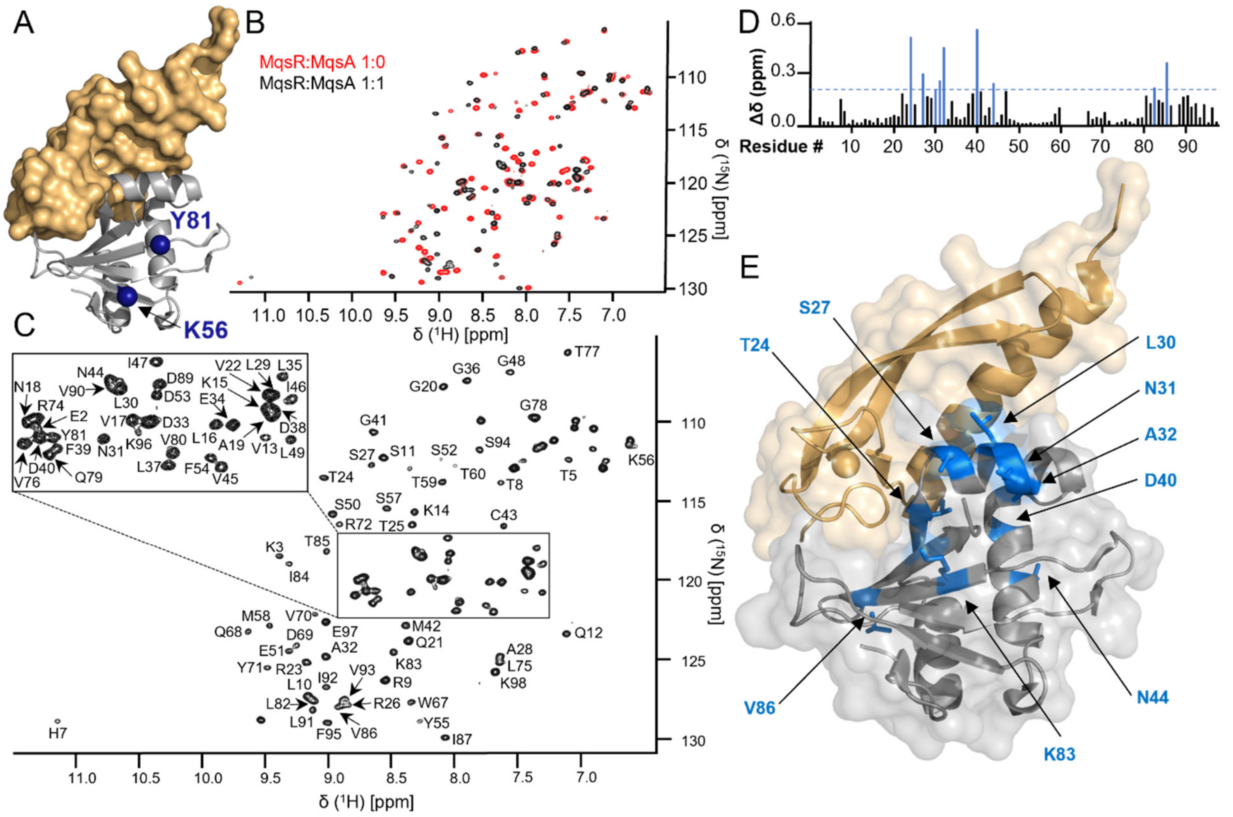

Figure S7: MqsA binds distally to the MqsR active site. *A.* The crystal structure of MqsA (orange surface) bound to MqsR (grey cartoon). The two active site residues Tyr81 and Lys56 are displayed in dark blue. *B.* Fully annotated 2D [^{1}H,^{15}N] HSQC spectrum of ^{15}N-MqsR titrated with MqsA$_{1\text{-}76}$. *C.* Reassigned peaks of MqsR in the bound MqsR:MqsA complex. *D.* CSPs for MqsR:MqsA. Residues with significant CSPs (>1.5σ_0; indicated by a dotted line) are indicated in blue. *E.* MqsR:MqsA$_{1\text{-}76}$ crystal structure with residues of MqsR that exhibit significant CSPs (>1.5σ_0) upon MqsA binding shown as sticks, colored blue and labeled.

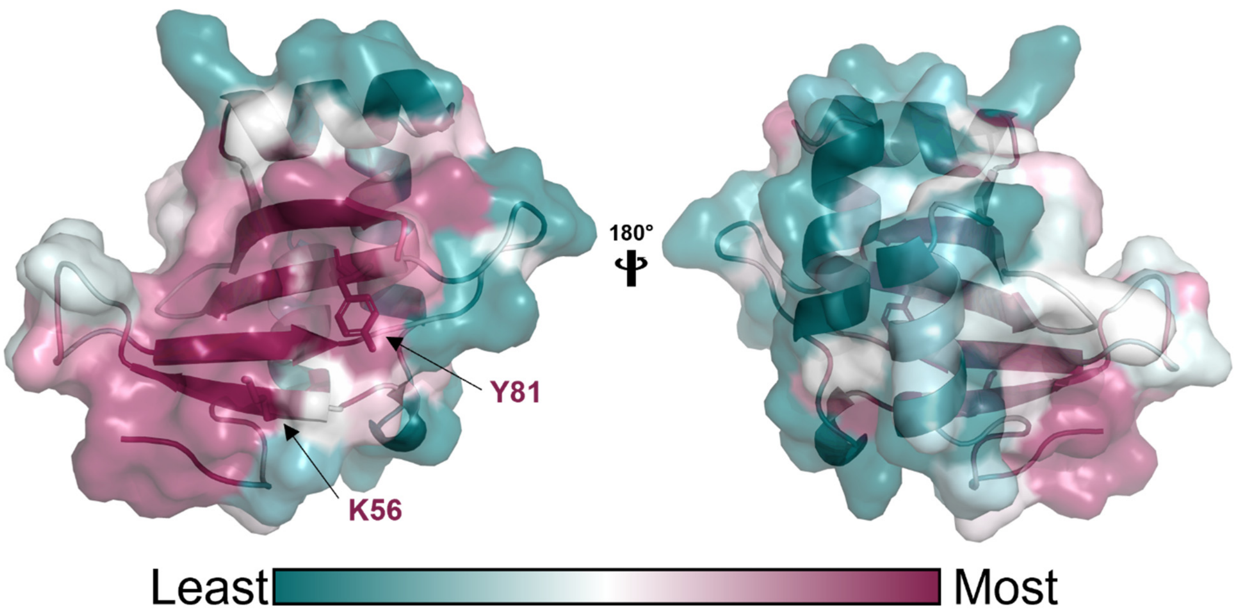

Figure S8: The active site of MqsR is the most well conserved region. Both Tyr81 and Lys56 sit in a well conserved region of MqsR that binds the substrate. Sequence conservation was determined using the ConSurf Server.

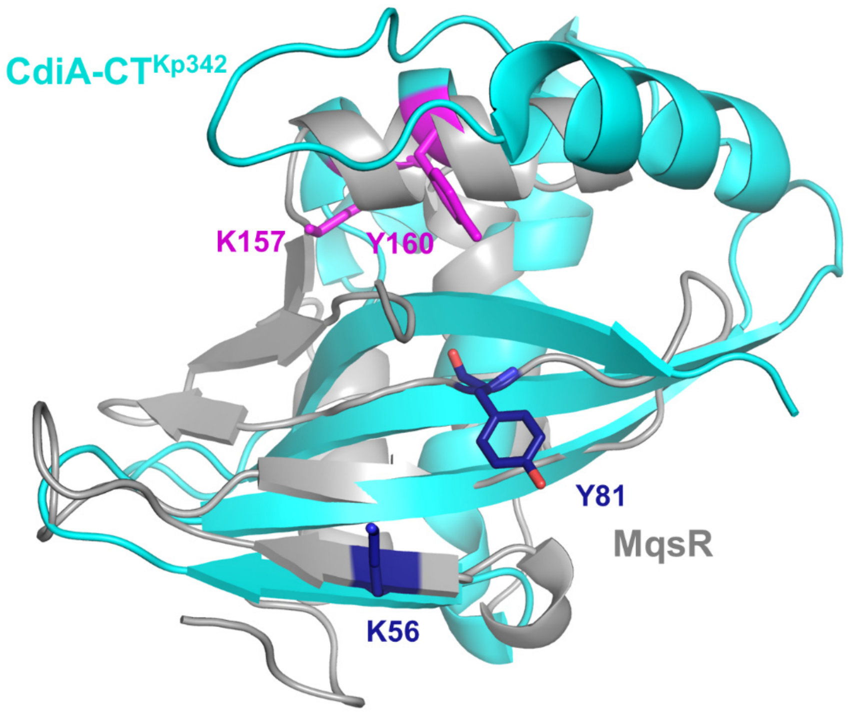

Figure S9. Overlay of MqsR and CdiA-CT^{Kp342}. MqsR (grey) and its catalytic residues Y81/K56 (dark blue, sticks) overlaid with CdiA-CT^{Kp342} (cyan, PDBID 3CP9) and its catalytic residue Y160/K157 (magenta, sticks). While both RNases share overall tertiary similarities (DALI Z-score of 5.6), and identical residues that are critical for catalysis (lys/tyr), the locations of the active sites differ.
